# Glucose repression can be alleviated by reducing glucose phosphorylation rate in *Saccharomyces cerevisiae*

**DOI:** 10.1038/s41598-018-20804-4

**Published:** 2018-02-08

**Authors:** Stephan Lane, Haiqing Xu, Eun Joong Oh, Heejin Kim, Anastashia Lesmana, Deokyeol Jeong, Guochang Zhang, Ching-Sung Tsai, Yong-Su Jin, Soo Rin Kim

**Affiliations:** 1 Carl Woese Institute for Genomic Biology, University of Illinois at Urbana-Champaign, Urbana, Illinois USA; 2 Department of Food Science and Human Nutrition, University of Illinois at Urbana-Champaign, Urbana, Illinois USA; 3 School of Food Science and Biotechnology, Kyungpook National University, Daegu, Republic of Korea; 4 Institute of Agricultural Science & Technology, Kyungpook National University, Daegu, Republic of Korea

## Abstract

Microorganisms commonly exhibit preferential glucose consumption and diauxic growth when cultured in mixtures of glucose and other sugars. Although various genetic perturbations have alleviated the effects of glucose repression on consumption of specific sugars, a broadly applicable mechanism remains unknown. Here, we report that a reduction in the rate of glucose phosphorylation alleviates the effects of glucose repression in *Saccharomyces cerevisiae*. Through adaptive evolution under a mixture of xylose and the glucose analog 2-deoxyglucose, we isolated a mutant strain capable of simultaneously consuming glucose and xylose. Genome sequencing of the evolved mutant followed by CRISPR/Cas9-based reverse engineering revealed that mutations in the glucose phosphorylating enzymes (Hxk1, Hxk2, Glk1) were sufficient to confer simultaneous glucose and xylose utilization. We then found that varying hexokinase expression with an inducible promoter led to the simultaneous utilization of glucose and xylose. Interestingly, no mutations in sugar transporters occurred during the evolution, and no specific transporter played an indispensable role in simultaneous sugar utilization. Additionally, we demonstrated that slowing glucose consumption also enabled simultaneous utilization of glucose and galactose. These results suggest that the rate of intracellular glucose phosphorylation is a decisive factor for metabolic regulations of mixed sugars.

## Introduction

The baker’s yeast *Saccharomyces cerevisiae* has long served as a model for studying glucose repression, the multi-layer process by which glucose is consumed before all other carbon sources^1,2^. A wide variety of interconnected mechanisms contribute to yeast’s ability to sense, respond, and optimize internal metabolism to preferentially consume glucose^3,4^. Transcriptional repressors such as Mig1, Cat8, and the Ssn6/Tup1 complex prevent transcription of glucose-repressed genes, such as those involved in gluconeogenesis and metabolism of alternative carbon sources^5–8^. The activities of these repressors are then mediated by kinases and phosphatases such as Snf1 and Glc7/Reg1, respectively^6,9,10^. Beyond these intracellular sensing mechanisms, membrane sensors such as Snf3 and Rgt2 allow yeast to sense extracellular sugar concentrations and internalize signals^11^. In sum, the *S. cerevisiae* glucose repression pathway is a complex network of signals and regulations comprising significant amounts of research and a continuously growing base of knowledge.

Recently, a new layer of glucose repression of galactose consumption has been reported to be linked to the kinetic properties of sugar transporters^12^. Because sugars compete for cellular uptake, relative transport efficiency between two sugars will depend on extracellular sugar concentrations as well as transporter affinities (K_m_ values) for each sugar. Consequently, it was reported that the extracellular sugar concentrations coupled with transporter substrate affinity determine the intracellular sugar concentrations^12^. As *GAL* gene expression is repressed by intracellular glucose via the MIG1 protein^13^ and activated by intracellular galactose through the GAL3 protein^3^, competition for transport can be considered an additional layer to glucose repression by affecting the intracellular accumulation of sugars^12^.

Similarly, in engineered yeast expressing a heterologous xylose assimilation pathway, glucose inhibits consumption of xylose by outcompeting xylose for uptake through hexose transporters^14–18^. Although there is no transcriptional regulation of the heterologous xylose pathway as observed in endogenous galactose metabolism, reduced intracellular accumulation of xylose due to transport inhibition serves as a bottleneck for downstream metabolism. In recent years, metabolic engineers working to produce biofuels and biochemicals have put significant efforts towards identifying transporter mutants with reduced or eliminated glucose inhibition^14,19–21^, increased xylose transport capabilities^22^, or enhanced stability of exceptional xylose transporters^23,24^.

These recent additions to the understanding of glucose repression have considered the kinetic properties of transporters exclusively as the outermost layer of glucose repression. However, by enabling simultaneous uptake of glucose and other carbon sources without any alteration to sugar transporters, we provide evidence that the prevalent model is incomplete. We first present a laboratory evolution leading to the isolation of mutants capable of simultaneously consuming glucose and xylose. Through genome analysis and various genetic perturbations, we show that the simultaneous consumption of glucose and xylose does not result from any mutations in sugar transporters and is instead a consequence of reduced glucose metabolic flux via mutations in hexokinases and glucokinase. In order to validate that glucose phosphorylation rate is a key determinant of glucose repression, we recreated this phenotype in the parental strain by tuning down the expression levels of hexokinases with an inducible promoter. We also show the generality of this model by demonstrating co-consumption of glucose and galactose via the same mechanism. Integrating these results, we propose a revised model of the outermost layer of glucose repression where the kinetic properties of transporters and intracellular metabolic fluxes together determine intracellular accumulation of sugars, effects on downstream regulations, and overall sugar consumption rates.

## Results

### Adaptive evolution for glucose derepression

**Figure 1:**
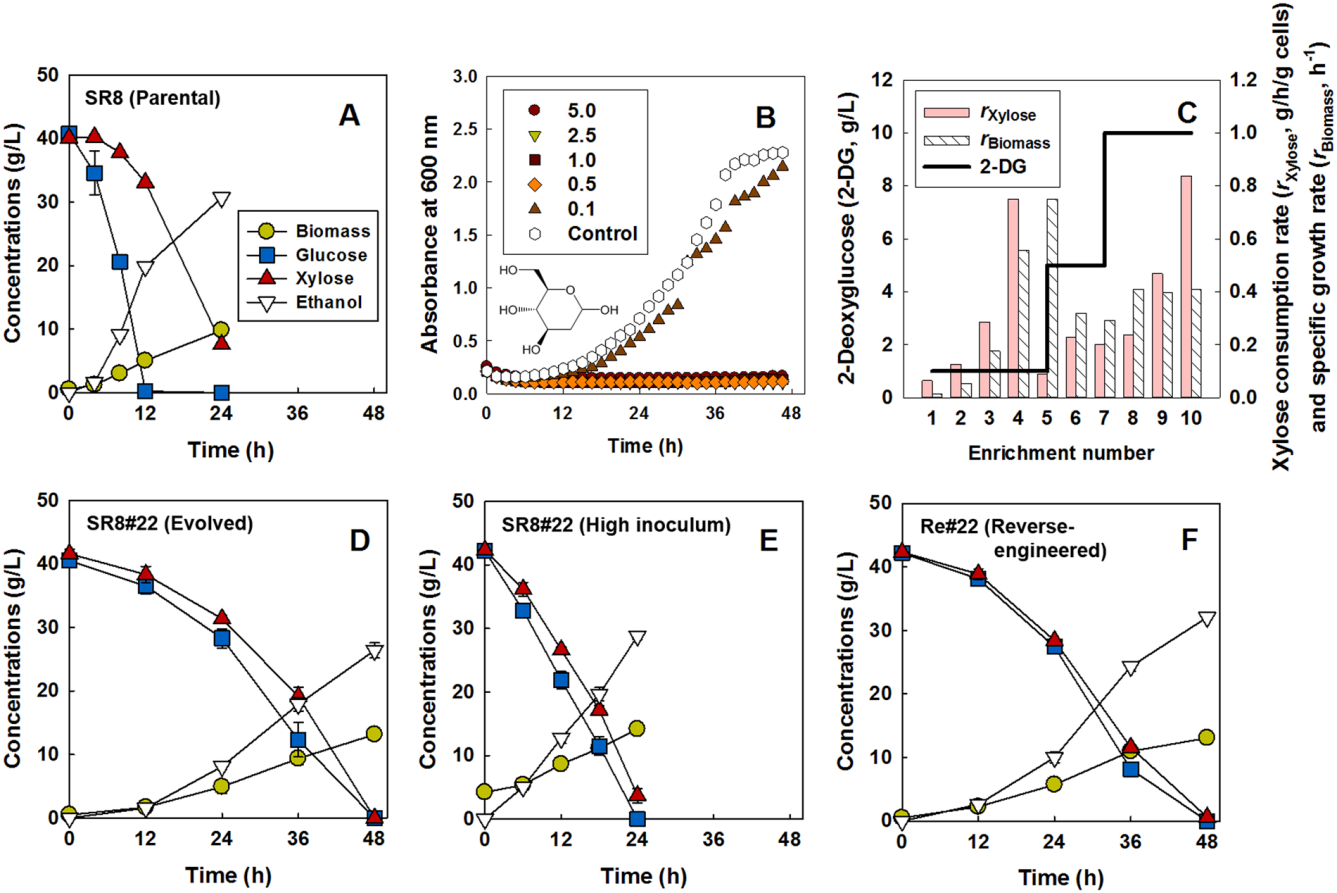
Adaptive evolution in 2-deoxyglucose and xylose leads to isolation of a mutant capable of co-consuming glucose and xylose. Co-fermentation of 40 g/L xylose and 40 g/L glucose in complex medium under oxygen-limited conditions by (**A**) the parental strain SR8, (**D**) the evolved strain SR8#22, and (**F**) the reverse-engineered strain Re#22 (SR8 *mGLK1 mHXK2 mHXK1*) with an initial cell concentration of 0.5 g/L, and (**E**) the SR8#22 strain with an initial cell concentration of 5 g/L. (**B**) Growth inhibition by 2-deoxyglucose in complex medium containing 40 g/L xylose. (**C**) Progressive improvement in the xylose consumption rates and the growth rates of the SR8 strain during serial subcultures in complex medium containing 40 g/L xylose and 2-deoxyglucose. The concentration of 2-deoxyglucose was periodically increased from 1 g/L to 10 g/L.

To overcome the repression of xylose metabolism by glucose in an engineered *S. cerevisiae* (Fig. 1A) expressing the genes (*XYL1*, *XYL2*, and *XYL3*) coding for xylose metabolic enzymes—xylose reductase, xylitol dehydrogenase, and xylulokinase—under strong and constitutive promoters^25^, we performed a laboratory evolution to generate mutants capable of simultaneous consumption of both sugars. To this end, we first screened non-metabolizable glucose analogues for their ability to repress xylose metabolism. Among six types of glucose analogues tested, 2-deoxyglucose (2-DG) at a concentration of more than 0.5 g/L exerted the most severe inhibition of cell growth of the xylose-metabolizing SR8 strain on xylose (Fig. 1B and Figure S1). Next, we evolved the SR8 strain in a medium containing 40 g/L of xylose and 0.5 g/L of 2-DG. Initially, xylose was consumed very slowly (Fig. 1C). However, after repeated sub-cultures under the same selective conditions, both the specific growth rate (h^−1^) and specific xylose consumption rate (g xylose/g cells/h) improved. By increasing the 2-DG concentration to 5 g/L and eventually to 10 g/L, we increased the selection pressure, which allowed the enrichment of mutants capable of metabolizing xylose with a high tolerance to 2-DG. After ten serial sub-cultures, we spread the evolved cells on glucose or xylose plates, and selected and compared 40 different colonies (Figure S2). Thirty-one colonies grew poorly on glucose and 9 colonies simultaneously consumed glucose and xylose (Figure S2C). For further study we chose one isolate, SR8#22, which exhibited simultaneous consumption of glucose and xylose. The evolved SR8#22 strain completed simultaneous consumption of sugars (40 g/L xylose and 40 g/L glucose) slower than the parental SR8 strain which consumes glucose and xylose sequentially (Fig. 1A,D). During co-consumption of sugars during mid-log phase, SR8 consumed glucose at 1.74 ± 0.29 g/g/h and xylose at 0.31 ± 0.02 g/g/h while the evolved SR8#22 consumed glucose at 0.23 ± 0.04 g/g/h and xylose at 0.19 ± 0.01 g/g/h (Table 1). The sugar co-consumption phenotype was not based on initial cell inoculum and was maintained when fermentations were initiated at higher cell density (Fig. 1E), suggesting the feasibility of overcoming the slow rate of co-fermentation through engineering approaches, such as cell recycling or immobilization. When using a single sugar, the SR8#22 strain exhibited a reduced glucose consumption rate compared with the parental strain, whereas the xylose consumption rate was maintained after the evolution (Figure S3). Additionally, expression of the genes in the pentose phosphate pathway were not substantially altered in the evolved SR8#22 as compared to the parental SR8 (Figure S4).

**Table 1:** Measurements of fermentation characteristics of strains in this study.

Strain	*r*_glucose_ (g/g/h)	*r*_xylose_ (g/g/h)	*P*_ethanol_ (g/g/h)	*Y*_ethanol_ (g/g)	Ethanol titer (g/L)
SR8 (Parental)	1.74 ± 0.29	0.31 ± 0.02	0.93 ± 0.02	0.42 ± 0.01	30.69 ± 0.39
SR8#22 (Evolved)	0.23 ± 0.04	0.19 ± 0.01	0.18 ± 0.02	0.32 ± 0.01	26.39 ± 1.2
SR8#22 *∆mGLK1*	0.14	0.20	0.12	0.33	23.44 ± 0.53
SR8#22 *∆mHXK1*	0.27	0.25	0.20	0.33	27.46 ± 0.14
SR8#22 *∆mHXK2*	0.23 ± 0.02	0.25 ± 0.02	0.16	0.35 ± 0.03	28.15 ± 0.88
SR8#22 pCYC1-mGLK1	0.16 ± 0.01	0.19	0.08	0.30 ± 0.01	9.45 ± 0.54
SR8#22 pTEF1-mGLK1	0.48 ± 0.02	0.16	0.25	0.33 ± 0.06	24.83 ± 1.08
SR8#22 pCCW12-mGLK1	0.65 ± 0.04	0.19 ± 0.02	0.35	0.32 ± 0.06	24.15 ± 1.02
SR8*∆*3iHXK2					
[Dox] = 0 µg/mL	0.04	0.18 ± 0.01	0.06	0.22	7.15 ± 0.01
[Dox] = 2 µg/mL	0.07	0.18	0.07	0.26 ± 0.01	9.76 ± 0.11
[Dox] = 4 µg/mL	0.21 ± 0.01	0.17 ± 0.01	0.15	0.34 ± 0.01	26.63 ± 0.27
[Dox] = 6 µg/mL	0.17 ± 0.01	0.16 ± 0.01	0.12	0.34	25.74 ± 0.28
[Dox] = 8 µg/mL	0.39 ± 0.02	0.16	0.22 ± 0.01	0.35 ± 0.02	29.1 ± 1.18
[Dox] = 10 µg/mL	0.42 ± 0.01	0.16	0.24 ± 0.01	0.33 ± 0.01	27.96 ± 0.62
[Dox] = 12 µg/mL	0.49 ± 0.02	0.17 ± 0.01	0.27 ± 0.01	0.31 ± 0.01	26.05 ± 1.14

Values are the average of biological duplicates with standard deviation. No standard deviation is shown when the value is below 0.01. *r*_glucose_, specific glucose consumption rate (g glucose/g dry cell weight/h); *r*_xylose_, specific xylose consumption rate (g xylose/g dry cell weight/h); *P*_ethanol_, specific productivity of ethanol (g ethanol/g dry cell weight/h); *Y*_ethanol_, ethanol yield (g ethanol/g consumed sugars). *r*_glucose_, *r*_xylose_, and *P*_ethanol_ are calculated in mid-exponential phase during co-consumption of glucose and xylose. *Y*_ethanol_ is calculated from the entirety of fermentation. Values were calculated from the fermentations shown in Fig. 1A (SR8), 1D (SR8#22), S8 (SR8#22 derivatives), 4, and S10 (SR8*∆*3iHXK2).

### Genome analysis of the evolved SR8#22 strain

To determine genetic changes responsible for allowing the evolved mutant to co-consume glucose and xylose simultaneously, the genome sequence of the SR8#22 strain was compared with that of the parental strain. Among 15 identified non-synonymous mutations, three SNPs in *GLK1, HXK2*, and *HXK1* were confirmed by Sanger sequencing (Table 2). Through backcrossing with the SR8 *MAT*a strain, three segregants were obtained exhibiting the evolved phenotype of the SR8#22 strain, sharing identical mutations in *GLK1, HXK2*, and *HXK1* (Table S1).

**Table 2:** Mutations identified in the evolved strain.

	*GLK1*	*HXK2*	*HXK1*
Nucleotide changes	265A > G	1364∆C	916T > C
Amino acid changes	Thr89Ala	Pro455fs	Ser306Pro
SR8#22 (Evolved)	**+**	**+**	**+**
SR8*mGLK1*	**+**	−	−
SR8*mGLK1mHXK2*	**+**	**+**	−
Re#22 (Reverse-engineered)	**+**	**+**	**+**

To confirm the result, the three mutations in the *GLK1* (265A > G)*, HXK2* (1364ΔC), and *HXK1* (916T > C) genes were sequentially introduced into the parental SR8 strain using CRISPR/Cas9 genome editing, yielding the SR8*mGLK1*, SR8*mGLK1mHXK2*, and SR8*mGLK1mHXK2mHXK1* (Re#22) strains (Table 2). Although the intermediate strains, SR8*mGLK1* and SR8*mGLK1mHXK2*, maintained sequential consumption of glucose and xylose (Figure S5), introduction of two mutations in hexokinase and one mutation in glucokinase (the Re#22 strain) regenerated the co-consumption phenotype (Fig. 1F) observed in the evolved mutant SR8#22 strain (Fig. 1D). These results demonstrate that the co-consumption observed in the SR8#22 strain is associated with all three mutations in the *GLK1, HXK2*, and *HXK1* genes.

### Molecular mechanisms of simultaneous co-fermentation

**Figure 2:**
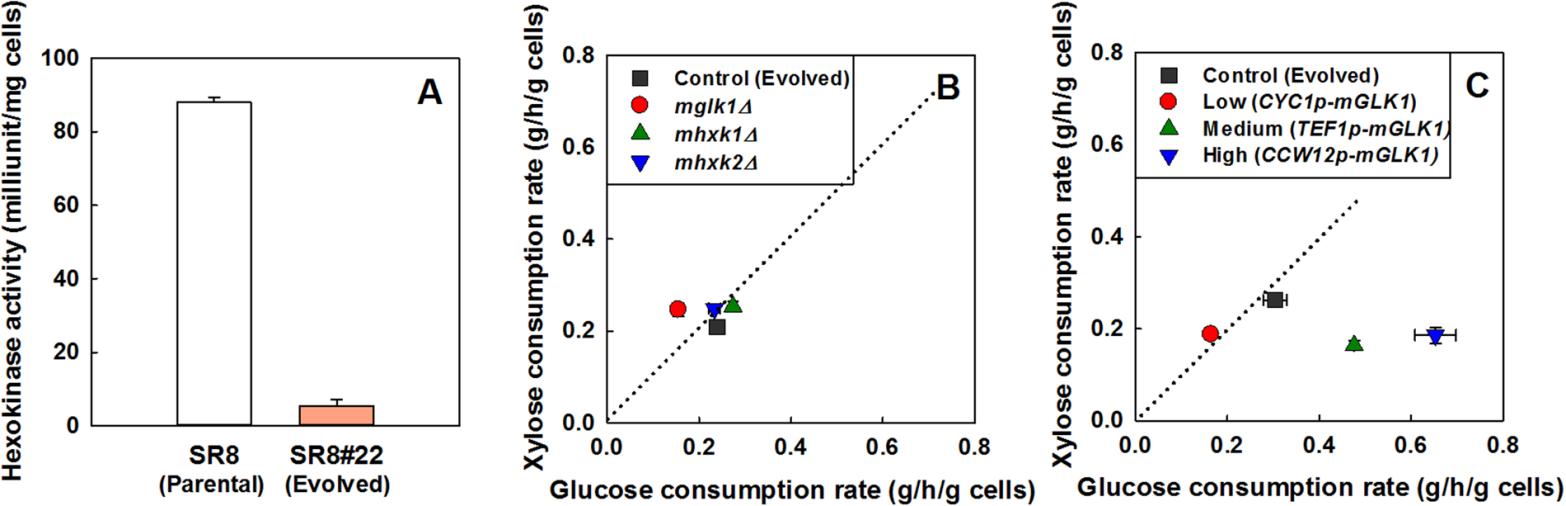
Reduced hexokinase activity is a critical determinant of mixed-sugar utilization. (**A**) *In vitro* hexokinase activity of the parental strain SR8 and the evolved strain SR8#22. (**B**,**C**) Specific consumption rates of glucose and xylose in a mixture of 40 g/L glucose and 40 g/L xylose by the hexokinase deletion mutants of the SR#22 strain and the promoter substitution mutants of the mutant *GLK1* gene in the SR8#22 strain: (**B**) the SR8#22 (control), SR8#22 *glk1*Δ, SR8#22 *hxk2*Δ, and SR8#22 *hxk1*Δ strains; and (**C**) the SR8#22 (control), SR8#22 *CYC1p*-*mGLK1* (low-strength promoter), SR8#22 *TEF1p*-*mGLK1* (medium-strength promoter), and SR8#22 *CCW12p*-*mGLK1* (high-strength promoter) strains. Specific consumption rates were calculated over a 12-hour period from two data points when cells were in mid-log phase and undergoing simultaneous consumption of glucose and xylose.

The *GLK1, HXK1*, and *HXK2* encode a glucokinase and two hexokinases, respectively, which initiate glycolysis by phosphorylating glucose. The mutations in the three genes, therefore, are likely related to the reduction in the glucose consumption rate of the SR8#22 strain. The SR8#22 strain had only 6% of *in vitro* hexokinase activity compared with that of the parental strain (Fig. 2A). Furthermore, protein structure analysis showed that the mutations in *GLK1* (Figure S6A), *HXK1* (Figure S6B), and *HXK2* (Figure S6C) were near the predicted ligand-binding sites. The Glk1p T89A mutation was in a loop between two β-strands and the most distant from the predicted ligand-binding site as compared to the mutations in Hxk1p and Hxk2p (Figure S6A). The Hxk1p S306P mutation was directly adjacent to the predicted glucose-binding site, in a loop separating two α-helices (Figure S6B). The 1364∆C mutation in the *HXK2* gene would lead to a frameshift mutation at amino acid 455. The affected residues were also located near the ligand-binding site and would affect an entire α-helix near the C-terminus of the protein (Figure S6C).

We also utilized RNA-seq to compare the expression of hexokinases across the parental SR8 and the evolved SR8#22 strains when cultured in glucose, xylose, or a mixture of glucose and xylose (Figure S7). We observed a significant increase in *GLK1* expression (*p* < 0.05) in the SR8#22 strain compared to the SR8 strain when cultured in glucose. Additionally, culturing on a mixture of glucose and xylose resulted in an increased expression of *HXK1* in the evolved SR8#22 as compared to parental SR8. As Hxk2p represses the expression of the *HXK1* and *GLK1* genes in the presence of glucose, this result may indicate that the Hxk2 protein had lost its ability to perform as a key regulator of the glucose repression pathway^26^. Based on these results, we hypothesized that the co-consumption observed by the SR8#22 strain might be caused from decreased overall hexokinase activity.

To confirm the above hypothesis, the necessity of the mutant *GLK1, HXK2*, and *HXK1* alleles in the SR8#22 strain was examined by individual gene deletion. While deletion of either *HXK1* or *HXK2* still allowed complete sugar consumption within 48 hours, deletion of *GLK1* reduced the overall efficiency of fermentation and nearly 30 g/L of sugars remained after 48 hours. Nonetheless, all single deletion mutants maintained the co-consumption phenotype of the SR8#22 strain (Fig. 2B and Figure S8A–C). This result suggests that the mutant glucokinase contributed most to the overall hexokinase activity of the SR8#22 strain.

Next, we investigated the effects of changing glucose consumption rates on mixed-sugar utilization in our evolved strain. We manipulated the expression levels of the mutant *GLK1* gene of the SR8#22 strain using a CRISPR/Cas9-based promoter substitution strategy, which was developed previously^27^. Alternative promoters with various strengths—strong (*CCW12p*), medium (*TEF1p*), and weak (*CYC1p*)—were introduced directly into upstream of the start codon of the mutant *GLK1* gene, resulting in the SR8#22-*CYC1p-mGLK1*, SR8#22-*TEF1p-mGLK1*, and SR8#22-*CCW12p-mGLK1* strains. Altered *mGLK1* expression levels led to varied glucose consumption rates in the three strains, whereas the xylose consumption rates remained similar in all mutants (Fig. 2C and Figure S8D–F). In other words, the xylose consumption rate was independent from the glucose consumption rate in the range we tested. This result suggests that intracellular glucokinase activity severely impacts consumption of mixed-sugars.

### Altered expression of sugar transporters in the evolved SR8#22 and their role in co-consumption of glucose and xylose

**Figure 3:**
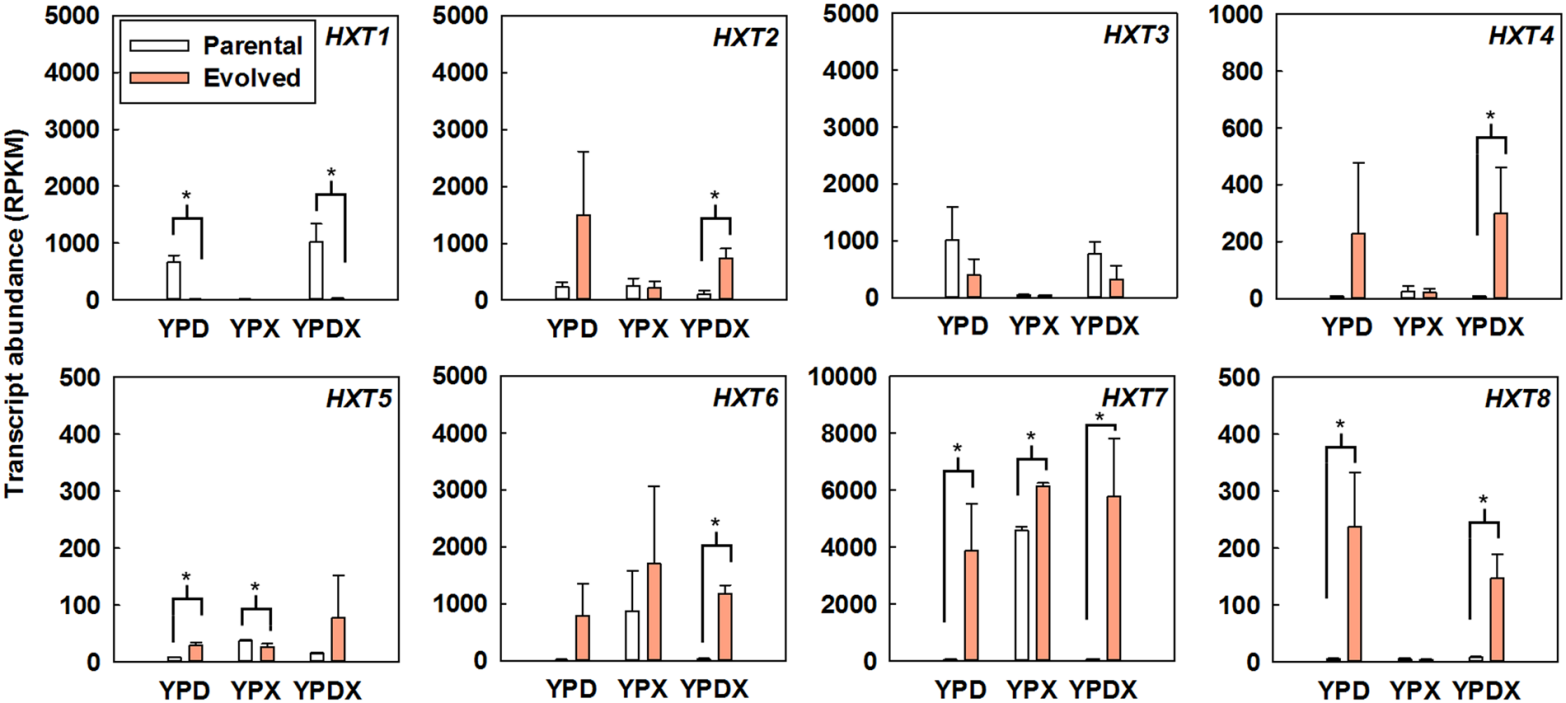
Comparison of expressions of sugar transporters in different sugar conditions. The parental SR8 strain and the evolved SR8#22 strain were cultured in YP medium containing 40 g/L glucose (YPD), YP medium containing 40 g/L xylose (YPX), and YP medium containing 40 g/L glucose and 40 g/L xylose (YPDX) at an initial OD of 0.1. Cells were grown to mid-exponential phase and RNA was extracted and quantified using RNA-seq as described in materials and methods. Among 18 hexose transporters,10 transporters with low expression levels (RPKM < 50) were not presented. A significant difference of *p* < 0.05 is indicated by a single asterisk. RPKM: reads per kilobase of transcript per million mapped reads.

A link between glycolytic flux and regulation of hexose transporters in yeast has been suggested^28^. Furthermore, it has been shown that xylose transport is inhibited by the presence of glucose^14,15^. Therefore, we sought to investigate changes in expression of sugar transporters and any potential effects on co-consumption of glucose and xylose through RNA-seq analysis. We first compared the expression profiles of sugar transporters in the parental SR8 and evolved SR8#22 strains when cultured with glucose, xylose, or a mixture of glucose and xylose (Fig. 3). The expression patterns of the two strains were different the most when cultured in a mixture of glucose and xylose (Fig. 3C); except for *HXT3*, all of the transporters were differentially expressed (*p* < 0.05). To determine effects of transporter deletion on co-fermentation capabilities, we individually deleted the five transporters expressed highest in the SR8#22 strain during co-consumption of glucose and xylose (*HXT2, HXT3*, *HXT4*, *HXT6*, and *HXT*7). Because of high sequence similarity (99% identity with only three single nucleotide polymorphisms), *HXT6* and *HXT*7 were deleted simultaneously. However, the deletion mutants of each transporter, SR8#22 *hxt2∆*, SR8#22 *hxt3∆*, SR8#22 *hxt4∆*, and SR8#22 *hxt6/7∆*, presented only marginal changes in the rate of mixed sugar consumption (Figure S9). This result confirms that no individual transporter is necessary for co-consumption and supports our original hypothesis of a glucose phosphorylation rate-based mechanism.

### Modulation of wild-type hexokinase expression

Although our previous results provide ample support for our hypothesis that the reduced rate of glucose phosphorylation might enable simultaneous co-utilization of glucose and xylose, our evolved SR8#22 nonetheless contains mutations in all three glucose phosphorylating enzymes. Therefore, we aimed to develop a system that can tightly control glucose phosphorylation rate without imposing mutations upon endogenous genes.

**Figure 4:**
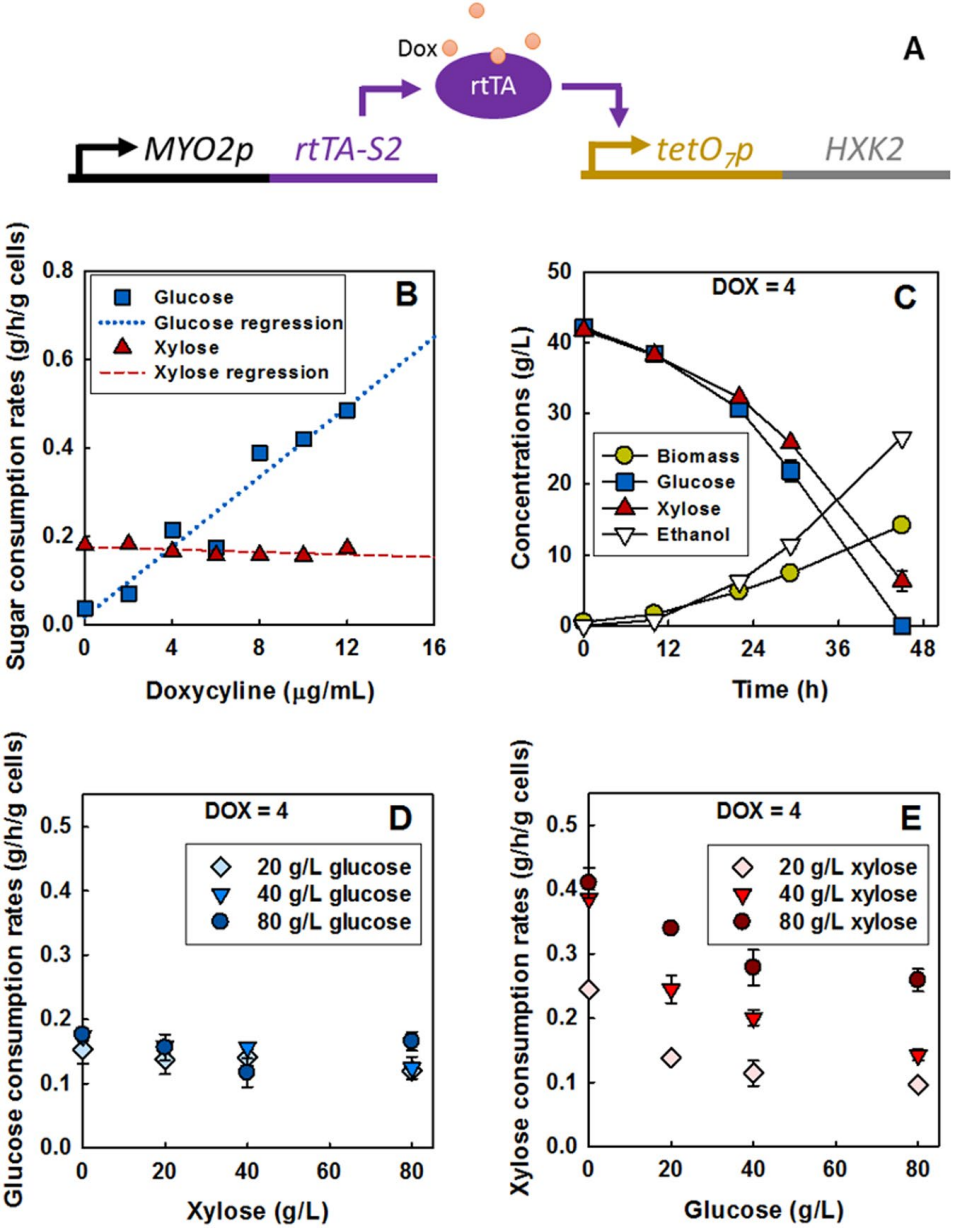
Extracellular sugar concentrations and intracellular hexokinase activity independently impact mixed-sugar utilization. (**A**) Scheme for controlling *HXK2* transcription using the doxycycline-controlled transactivator rtTA-S2. The regulatory system was introduced into the hexokinase null mutant (SR8 *glk1*∆, *hxk2*∆, *hxk1*∆), yielding the SR8∆3iHXK2 strain. (**B**) Glucose and xylose consumption rates over 12 h in a mixture of 40 g/L glucose and 40 g/L xylose by the SR8∆3iHXK2 strain with 0–12 μg/mL doxycycline. With 4 μg/mL doxycycline, consumption rates of the two sugars were identical. (**C**) Fermentation profiles of the SR8∆3iHXK2 strain in a mixture of 40 g/L glucose and 40 g/L xylose with 4 μg/mL doxycycline. (**D**) The effect of xylose concentration on glucose consumption rates, and (**E**) the effect of glucose concentration on xylose consumption rates at a constant doxycycline concentration of 4 μg/mL.

Using a hexokinase null mutant of the SR8 strain (*hxk1Δ*, *hxk2Δ*, and *glk1Δ*), we developed a doxycycline-mediated titratable expression system^29^ of the *HXK2* gene, yielding the SR8*∆*3iHXK2 strain (see Materials and Methods). As illustrated in Fig. 4A, the wild-type *HXK2* gene is expressed under the control of the *tetO*_*7*_ promoter, which is activated by doxycycline in a concentration-dependent manner. The glucose consumption rate of the SR8*∆*3iHXK2 strain increased proportionally to the extracellular doxycycline concentration (0–12 μg/mL) in a mixture of glucose and xylose (Fig. 4B and Figure S10). However, the xylose consumption rate stayed largely constant across the entire induction range tested (Fig. 4B). With 4–6 μg/mL doxycycline, the glucose consumption rate was similar to that of xylose (0.2 g/h/g cells), and the two sugars were co-consumed equally (Fig. 4C). In contrast, inducing hexokinases at above or below 4–6 µg/mL doxycycline resulted in an unbalanced consumption of the two sugars (Figure S10). These results demonstrate that regulation of hexokinase activity on its own impacts mixed-sugar utilization.

As prior reports have shown severe reduction of xylose consumption due to glucose inhibition of xylose transport, we next investigated the effects of changing extracellular sugar concentrations while maintaining a constant level of hexokinase induction. We therefore induced hexokinase expression with 4 µg/mL of doxycycline, a level which previously led to nearly equal consumption of both sugars, and initiated cultures with varied extracellular sugar concentrations. The rate of glucose consumption generally showed minor variations as extracellular sugar concentrations were varied (Fig. 4D). However, xylose consumption was substantially affected by variations in extracellular sugar concentrations (Fig. 4E). The xylose consumption rate significantly increased as xylose concentrations were raised (*p* < 0.05): 20 g/L of xylose led to a consumption rate of 0.23 ± 0.003 g/g/h, while 80 g/L of xylose led to a consumption rate of 0.38 ± 0.02 g/g/h. Additionally, with 20 g/L of xylose, increasing the extracellular glucose concentration from 0 g/L to 80 g/L led to a significant decrease in xylose consumption rate from 0.23 ± 0.003 g/g/h to 0.09 ± 0.01 g/g/h (*p* < 0.05). These results demonstrate that both extracellular sugar concentrations and intracellular hexokinase activity independently impact mixed-sugar utilization.

### Co-consumption of glucose and galactose by modulating hexokinase activity

**Figure 5:**
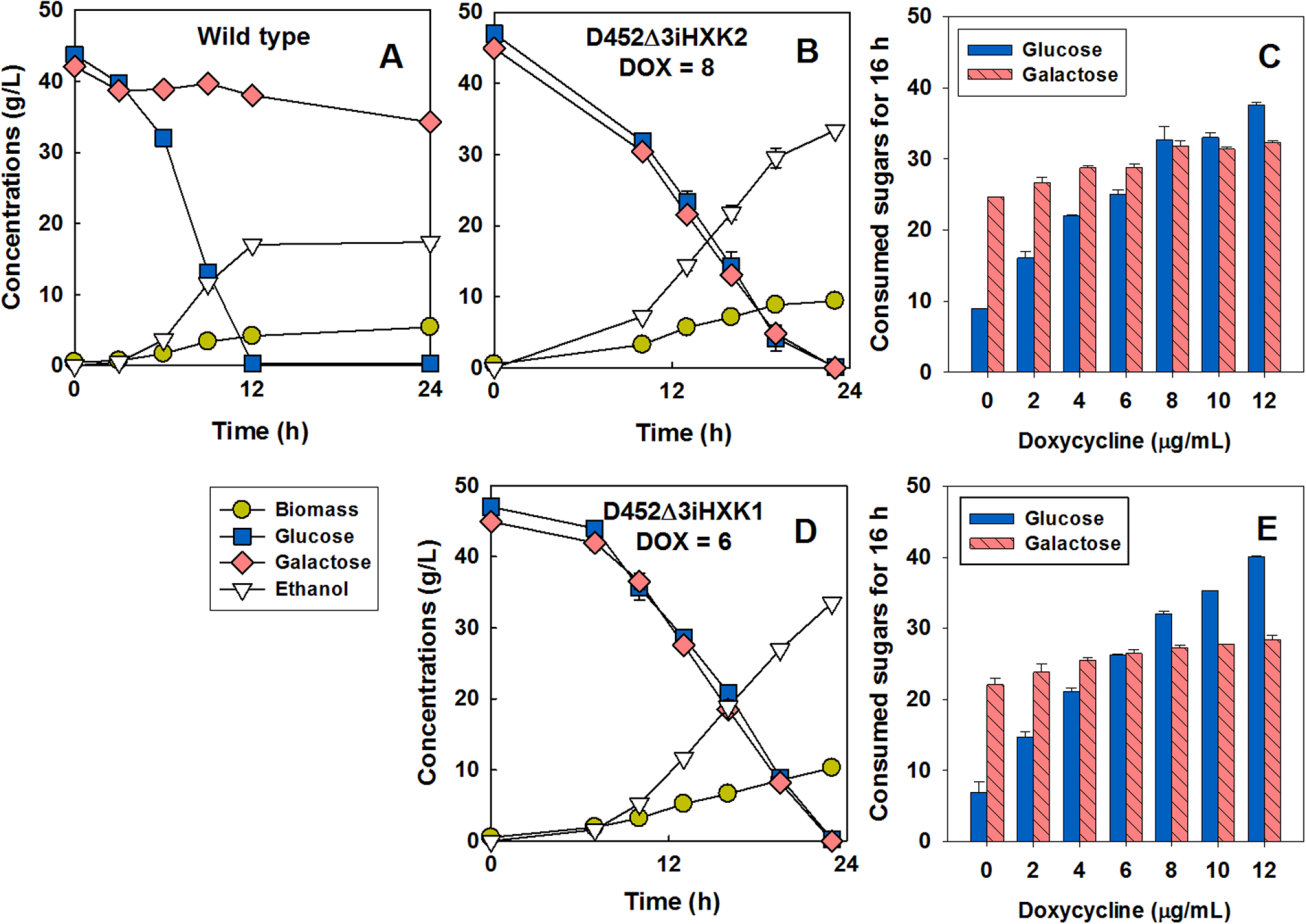
Hexokinase activity impacts mixed-sugar fermentation of glucose and galactose. Fermentation profiles in a mixture of glucose and galactose by the wild-type strain (D452-2) expressing a control vector (pRS403) (**A**), the D452∆3iHXK2 strain expressing the inducible *HXK2* gene with 8 μg/mL doxycycline (**B**), and the D452∆3iHXK1 strain expressing the inducible *HXK1* gene with 6 μg/mL doxycycline (**D**). Both strains were constructed using the hexokinase null mutant of the D452-2 strain (D452-2 *glk1*∆, *hxk2*∆, *hxk1*∆). The sugars consumed over 16 h in a mixture of glucose and galactose by the D452∆3iHXK2 strain (**C**) and the D452∆3iHXK1 strain (**E**) with 0–12 μg/mL doxycycline. Arrows indicate when the consumption rates of the two sugars were identical.

Thus far, our results have shown that transport inhibition and the rate that glucose enters glycolysis have independent effects on glucose repression. Furthermore, transport inhibition has recently been emphasized as a major player in glucose repression of galactose by way of determining the intracellular accumulation of sugars and downstream effects on transcriptional regulation of galactose metabolic genes^3,12,13^. We thus hypothesized that hexokinase activity may again play a role independent from transport inhibition by altering the accumulation of intracellular sugars. In our parental strain, galactose metabolism is strongly repressed by the presence of glucose, leading to a sequential utilization of the two sugars (Fig. 5A). To avoid any unknown effects on galactose metabolism caused by genetic changes in the engineered SR8 strain^25^, we used the D452-2 strain, the wild-type origin of the SR8 strain^30^, for this portion of the study. The three endogenous hexokinases were deleted in D452-2 strain, and doxycycline-regulated expression of the *HXK2* gene was introduced, resulting in the D452*∆*3iHXK2 strain (see Supplementary Information).

In the D452*∆*3iHXK2 strain, the glucose consumption rates were tightly regulated by the expression levels of *HXK2* in a mixture of glucose and galactose (Fig. 5B,C and Figure S11, Table S2), which was consistent with the SR8∆3iHXK2 strain (Fig. 4B). However, the galactose consumption rates remained constant throughout the doxycycline concentrations we tested (Fig. 5C and Figure S11). Thus, we observed a balanced consumption of glucose and galactose when hexokinase expression was induced with 8 µg/mL doxycycline (Fig. 5B). Moreover, when the doxycycline-dependent expression system was constructed with the *HXK1* gene instead of *HXK2*, a similar pattern of the balanced consumption was achieved with 6 µg/mL doxycycline (Fig. 5D,E, and Figure S12, Table S2). Similar to the observations with mixtures of glucose and xylose, the simultaneous consumption could be tilted in favor of either glucose or galactose by increasing or decreasing the doxycycline concentration, respectively. In addition to our results with mixtures of glucose and xylose, we again found that the rate glucose enters glycolysis impacts mixed-sugar fermentation of glucose and galactose.

## Discussion

### Transport kinetics and intracellular metabolic flux together constitute the outermost layer of glucose repression

Previous reports have highlighted transporter preference for glucose as the outermost layer of glucose repression and the first cause of preferential consumption of glucose over both galactose^12^ and xylose^15^. However, we have shown that an adaptive evolution can lead to isolation of a mutant capable of simultaneous uptake of glucose and xylose without any alteration of transporters, that this phenotype can be recreated rationally by limiting expression of hexokinases, and that the same genetic perturbations can lead to simultaneous uptake of glucose and galactose. Taken together, these results demonstrate that the transporter-centric model is incomplete and that a complete understanding of the effects of transporter inhibition in glucose repression can only be achieved when considered in combination with intracellular metabolic fluxes (Figure S13). On top of this, galactose metabolic genes are regulated by intracellular glucose and galactose repressing and activating transcription, respectively^31^. By limiting the rate of glucose phosphorylation, the accumulation of intracellular glucose is increased, the process of ratio sensing through transporter preference is altered, and the subsequent downstream regulatory effects will be changed. These results open the possibility that endogenous inhibitors of hexokinase, such as trehalose-6-phosphate^32,33^, may play a role in mixed sugar utilization and the ability to modulate response to sugar ratios.

Although yeast is highly specialized for preferential consumption of glucose, it has been shown capable of converting its metabolism to resemble a metabolic generalist through the [GAR+] prion^34^. While there is a growing base of knowledge on generating this prion, natural induction of the prion^35,36^, and phenotypes of yeast harboring the prion^37^, the exact metabolic changes and underlying network of molecular interactions are still poorly understood. After conversion to a metabolic generalist, *S. cerevisiae* exhibits an enhanced growth rate in mixed sugars when the fraction of glucose is below 50%, but reduced growth rate when the fraction of glucose is above 50%^34^, suggesting a reduced glucose consumption rate. It is feasible that modulation of glucose flux contributes to mixed-sugar utilization during the switch between a metabolic specialist and generalist. However, more investigation is required to understand the exact effect of the [GAR+] prion on glucose consumption rates and its connections with the known variations in growth rates during mixed-sugar consumption.

### The Snf3/Rgt2 glucose sensing pathway can be overruled by reduced glycolytic flux in determining hexose transporter expression

Snf3 and Rgt2 are membrane glucose sensors which internalize information about extracellular glucose concentrations^11^. In addition to playing a role in regulating the growth rate of yeast^38^, the low-affinity Rgt2 and high-affinity Snf3 initiate signal cascades ending in regulation of yeast glucose transporters^39^. They allow the yeast cell to sense and respond to high levels of glucose by expressing low-affinity glucose transporters, or in contrast, expressing high-affinity glucose transporters in response to low glucose concentrations. Further illustrating the tight regulations on yeast membrane sugar transport, non-optimal sugar transporters are rapidly removed in a process known as endocytosis and degraded in the vacuole^40^. In response to high levels of glucose, Hxt2^41^, Hxt6^42,43^, and Hxt7^44^ all undergo endocytosis and vacuolar degradation. On the other hand, Hxt1^45^ and Hxt3^46^ are rapidly internalized and degraded in response to glucose starvation. Furthermore, even the glucose sensors Snf3 and Rgt2 are degraded in high and low concentrations of glucose, respectively^47^.

Interestingly, we found that the evolved SR8#22 strain with reduced glucose phosphorylating rate had different expression patterns of sugar transporters from those of the parental SR8 strain (Fig. 3). Specifically, when cultured with high (40 g/L) initial glucose, the parental strain predominantly expressed the low-affinity glucose transporters Hxt1 and Hxt3, while the evolved mutant highly expressed the high-affinity glucose transporters Hxt2, Hxt6, and Hxt7 (Fig. 3). Although the evolved mutant SR8#22 contained no alterations to the Snf3/Rgt2 glucose-sensing pathway, a reduction in the glucose consumption rate was sufficient to induce changes in transporter expression. This observation lends support to previous reports of a link between glycolytic flux and the membrane composition of sugar transporters^28,48^. These results indicate that the regulation by the Snf3/Rgt2 pathway on transporter expression can be superseded by a reduction in glycolytic flux.

However, it is known that there is significant crosstalk between the Snf3/Rgt2 glucose induction and the Snf1-Mig1 glucose repression pathways^49^. There is also evidence that the Snf1 kinase may be regulated by glucose-6-phosphate^50,51^, which is likely present in decreased concentrations in our hexokinase-limited strains. It is thus possible that these two interconnected regulatory pathways only allow high expression of high-affinity glucose transporters when glucose-6-phosphate is at high levels and the Snf3/Rgt2 glucose induction pathway is activated.

### Co-consumption of sugars may afford unique opportunities in biotechnology

Thus far, orthogonal metabolism has only sparingly been used to enhance bioconversion processes despite its significant potential to expand capabilities of metabolic engineers^52^. Further, while most bioconversion processes rely on using one sugar for both cell growth and target molecule production, co-consumption of sugars allows the efficient use of flux partitioning strategies such that one carbon source is used for cell maintenance while the other carbon source is directed towards target production. For example, our evolved SR8#22 co-utilized glucose and xylose for cell growth with ethanol as the end metabolite. However, deletion of the heterologous xylitol dehydrogenase *XYL2* enables the conversion of xylose into xylitol while glucose is utilized as a carbon source for cell growth and maintenance. When *XYL2* is deleted in strain SR8 and the evolved SR8#22, xylitol production is enhanced through co-consumption of sugars (Figure S14).

Significant efforts have also been put towards creation of flux valves for enhancing production of target molecules^53–55^. In these scenarios, glycolytic flux is reduced to allow partitioning of flux off towards production of target molecules. Nonetheless, a balance must always be struck between maintaining sufficient glycolytic flux for cell growth and adequate flux for economical production of target molecules. To combat this problem, recently a dynamic flux valve was introduced which rapidly redirects flux towards target molecule production once cell growth has reached a certain threshold^56^. However, it may be simpler and more advantageous for sugar co-consumption to be combined with flux valves and orthogonal metabolism to further expand the possibilities within metabolic engineering for production of valuable biomolecules.

It has also recently been shown that weakening glycolysis can prove useful in destabilizing *Escherichia coli* substrate channeling^57^, a phenomenon wherein protein complexes directly channel substrates along desired metabolic pathways. Substrate channeling can decrease the chance for metabolic pathway intermediates to be directed towards engineered or heterologous pathways and may decrease yields, productivities and titers of desired products^58^. Additionally, there is some evidence for substrate channeling effects in the yeast pentose phosphate pathway^59^ and tricarboxylic acid cycle^60–62^. The methodology proposed here may enable increased production of target molecules by removing rigidity in pathways which may be subject to substrate channeling effects.

It is important to note that the particular methodology employed in this paper is very generalizable, as shown by our broad results with glucose/xylose and glucose/galactose mixtures. Depending on the goal of a particular flux partitioning or orthogonal metabolism effort, different sets of carbon sources may be desirable. The broad results presented in this report indicate that this mechanism could be further employed for co-consumption of glucose and other industrially-relevant carbon sources such as arabinose^63,64^ and 4-deoxy-l-erythro-5-hexoseulose urinate^65^.

## Methods

### Culture conditions

All plasmids and strains used in this study are listed in Table S3. Preculture was performed aerobically at 30 °C for 36 h in 5 mL of YP medium (10 g/L yeast extract, 20 g/L Bacto peptone) with 20 g/L of glucose except for the mixed sugar fermentations, which included 40 g/L of the appropriate carbon source; i.e. for fermentation of glucose/xylose mixture, cells were precultured in xylose, whereas for fermentation of glucose/galactose mixtures, cells were precultured in galactose. For hexokinase induction, the preculture medium was supplemented with doxycycline at the same concentration as in the main fermentation. For the main fermentations, the initial cell densities were adjusted to OD_600_ = 1 (optical density at 600 nm), which was 0.47 g/L dry cell weight. Fermentations were performed in 125 mL flasks containing 25 mL medium with an initial pH of 6.3 at 30 °C and 100 rpm.

### Analytical techniques

We used a Bioscreen C plate reader system (Growth Curves USA, Piscataway, NJ, USA) to monitoring cell growth under the presence of a type of glucose analogue and xylose, as previously described^25^. Biomass was calculated from the OD_600_ measured using a Biomate 5 UV-visible spectrophotometer (Fisher, NY, USA). We used a high-performance liquid chromatography (HPLC) system (Agilent, Santa Clara, CA, USA) with a Rezex RCM- Monosaccharide Ca+2 (8%) column (Phenomenex Inc., Torrance, CA, USA) to measure sugar concentrations, and used a Rezex ROA-Organic Acid H+ (8%) column (Phenomenex Inc.) to quantify ethanol concentrations.

### Adaptive evolution

Adaptive evolution of the SR8 strain was performed in YP medium containing 40 g/L xylose and 1 g/L 2-deoxyglucose. Starting with OD_600_ = 1, the cell concentration was monitored every 24 h. When the OD_600_ = 5, the cells were transferred to a new medium with initial OD_600_ = 1. These serial subcultures were performed with a gradual increase of 2-deoxyglucose concentration to 5 g/L, and ultimately 10 g/L. Forty single colonies were isolated from the 10th culture, and their phenotypes were evaluated.

### Genome sequencing and RNA-seq

Genome sequencing and SNP discovery of evolved mutants were performed as previously described^25^. RNA extraction, RNA-seq, and data analysis of sugar transporters in different sugar conditions were performed as previously described^66^.

### Strain engineering

A detailed description of the strain engineering methods is provided in the Supplementary Information. In short, the reverse engineering and the promoter substitution of the SR8#22 strain were performed by CRISPR/Cas9-based genome engineering^27,66,67^. We used both CRISPR/Cas9 genome engineering and the Cre/Loxp system^68,69^ for gene deletion in the SR8#22 strain.

### Doxycycline-controlled system of hexokinase expression

The rtTA(S2) variant^29^ was employed under the control of the yeast *MYO2* promoter^70^. The rtTA(S2) variant attached to the yeast *CYC1* terminator was synthesized and cloned into the pRS406 plasmid using SpeI and NotI restriction enzymes. The *MYO2* promoter was then amplified from yeast genomic DNA and cloned into the plasmid upstream of the rtTA(S2)-*CYC1t* cassette to create plasmid pRS403-rtTA. The doxycycline-inducible expression plasmids were created by ligating pRS403 with the *tetO*_7_-GFP expression cassette amplified from the pFA6a-kanMX-*tetO*_7_-*CYC1p*-GFP plasmid, which was a gift from Michael Nick Boddy (Addgene Plasmid #41025)^71^. The *GFP* gene was then removed and replaced with a multi-cloning site to create plasmid pRS403-*tetO*_7_, into which *HXK2* and *HXK1* were cloned to create plasmids pRS403-*tetO*_7_-*HXK2* and pRS403-*tetO*_7_-*HXK1*, respectively.

To create strain SR8*∆*3iHXK2, we began with a quadruple auxotroph (*his*∆, *leu*∆, *trp*∆, *ura*∆) version of SR8, and disabled glucose utilization by deleting the three endogenous hexokinases *GLK1, HXK1*, and *HXK2* to yield strain SR8*∆*3 (see Supplementary Information). We next genome-integrated the pRS406-rtTA and the pRS403-*tetO*_*7*_-*HXK2* plasmids into the SR8*∆*3 strain at the *URA3* and *HIS3* loci, respectively, resulting in the SR8*∆*3iHXK2 strain. Please see the Supplementary Information for the construction of the D452*∆*3iHXK2 and the D452*∆*3iHXK1 strains.

### Hexokinase activity assay

The cells were precultured in YP-galactose, and cell densities were adjusted to an approximate OD_600_ of 0.1 (0.047 g/L dry cell weight). The cells were grown in 50-mL flasks containing 10 mL YP medium with 20 g/L galactose at 30 °C and 100 rpm. When the cells reached exponential growth (OD_600_ = 1), they were harvested from the 0.5-mL culture and analyzed using a Hexokinase Colorimetric Assay Kit (St. Louis, MO, USA), following the manufacturer’s instructions.

## Electronic supplementary material

Supporting information
