# Supplementary material for: Glucose repression can be alleviated by reducing glucose phosphorylation rate in Saccharomyces cerevisiae
Source: Sci Rep. 2018 Feb 8;8:2613. doi: 10.1038/s41598-018-20804-4 (PMC5805702; doi:10.1038/s41598-018-20804-4)
Supplement: Supplementary file 1 — Supporting information [file 41598_2018_20804_MOESM1_ESM.docx]

**Glucose repression can be alleviated by reducing glucose phosphorylation rate in *Saccharomyces cerevisiae***

Stephan Lane^a,b,†^, Haiqing Xu^a,b,†^, Eun Joong Oh^a,b^, Heejin Kim^a,b^, Anastashia Lesmana^a,b^, Deokyeol Jeong^c^, Guochang Zhang^a,b^, Ching-Sung Tsai^a,b^, Yong-Su Jin^a,b,*^, and Soo Rin Kim^c,d,*^

^a^Carl Woese Institute for Genomic Biology, University of Illinois at Urbana-Champaign, Urbana, Illinois, USA

^b^Department of Food Science and Human Nutrition, University of Illinois at Urbana-Champaign, Urbana, Illinois, USA

^c^School of Food Science and Biotechnology, Kyungpook National University, Daegu, Republic of Korea

^d^Institute of Agricultural Science & Technology, Kyungpook National University, Daegu, Republic of Korea

*Corresponding authors: Yong-Su Jin ([ysjin@illinois.edu](mailto:ysjin@illinois.edu)) and Soo Rin Kim ([soorinkim@knu.ac.kr](mailto:soorinkim@knu.ac.kr))

† These authors made equal contributions

**Supplementary Methods**

**Reverse engineering of the evolved SR8#22 strain by CRISPR/Cas9**

The three mutations in the SR8#22 strain, *mGLK1* (265A>G), *mHXK2* (1364ΔC), and *mHXK1* (916T>C), were sequentially introduced to the parental SR8 strain through CRISPR/Cas9 genome engineering, which has been described previously ([1](#_ENREF_1), [2](#_ENREF_2)). First, the SR8 strain was transformed with the pRS41N-Cas9 plasmid and selected on YPD agar medium supplemented with 100 μg/mL nourseothricin sulfate (Gold Biotechnology, St. Louis, USA). Second, guide RNA plasmids were cloned with specific target sequences (20 bp) of the *GLK1*, *HXK2*, and *HXK1* genes (Table S4) on either the pRS42K or pRS42H plasmids ([1](#_ENREF_1)), yielding the pRS42K-gRNA-GLK1.1, pRS42H-gRNA-HXK2.1, and pRS42K-gRNA-HXK1.1 plasmids. Third, donor DNA fragments with the desired mutation were generated by PCR of the SOO459/SOO460 primers (Table S5). Next, the SR8 strain expressing the pRS41N-Cas9 plasmid was co-transformed with the pRS42K-gRNA-GLK1.1 plasmid and the donor DNA fragments, and the resulting transformants were selected on YPD agar medium supplemented with both 100 μg/mL nourseothricin sulfate and 200 μg/mL G-418 sulfate (Gold Biotechnology). After colony PCR and Sanger sequencing with the SOO8/SOO9 primers, the transformants were confirmed as the SR8*mGLK1* strain.

Following the same procedure, the second mutation, *mHXK2* (1364ΔC), was introduced to the SR8*mGLK1* strain using different guide RNA plasmid and donor DNA fragments; i.e., the pRS42H-gRNA-HXK2.1 plasmid and the PCR product of the SOO510/SOO511 primers. YPD agar medium supplemented with both 100 μg/mL nourseothricin sulfate and 150 μg/mL hygromycin B (Gold Biotechnology) was used to select the transformants, and confirmation was carried out using the SOO170/SOO171 primers.

Finally, the third mutation, *mHXK1* (916T>C), was introduced to construct the SR8*mGLK1mHXK2mHXK1* strain (Re#22) using the pRS42K-gRNA-HXK1.1 plasmid, the SOO546/SOO547 primers, and the SOO4/SOO5 primers.

**Gene deletion of the evolved SR8#22 strain**

The individual deletion of mutant glucokinase gene (*mglk1*∆, *mhxk2*∆, and *mhxk1*∆) and sugar transporter genes (*hxt3*∆*, hxt4*∆*,* and *hxt6/7*∆) of the SR8#22 strain was performed using a dominant drug resistance cassette ([3](#_ENREF_3), [4](#_ENREF_4)) except for *hxt2*∆ which was done by CRISPR/Cas9 genome engineering as described above.

To create the deletion mutants, the SOO252/SOO253 primers (*mglk1::loxP*), the Jin2335/Jin2337 primers (*hxt3::loxP*), the Jin2337/Jin2338 primers (*hxt4::loxP*), and the SOO401/SOO406 primers (*hxt6/7::loxP*) were used to PCR-amplify deletion cassettes from the pUG74 plasmid. For the SOO179/SOO180 primers (*mhxk2*::natMX) and the SOO142/SOO143 primers (*mhxk1*::natMX), pAG25 plasmid was used as a template. The SR8#22 strain was transformed with these deletion cassettes individually, and the transformants were selected on an YP agar plate containing 40 g/L Xylose and 100 μg/mL nourseothricin sulfate. Each deletion mutant was confirmed by a positive band from colony PCR using the following primer sets: SOO148/SOO225 (*mglk1::loxP*), SOO148/Jin2343 (*hxt3::loxP*), SOO148/Jin2344 (*hxt4::loxP*), and SOO148/SOO407 (*hxt6/7::loxP*), SOO187/SOO21(*mhxk2*::natMX), and SOO187/Jin687(*mhxk1*::natMX).

For the clean deletion of *HXT2* (*hxt2*∆)*,* the SR8#22 strain expressing the pRS41N-Cas9 plasmid was co-transformed with the pRS42H-gRNA-HXT2.1 plasmid and the donor DNA fragments generated by the Jin3266/Jin3267 primers. The resulting transformants were selected on YPD agar medium supplemented with both 100 μg/mL nourseothricin sulfate and 150 μg/mL hygromycin B. The deletion was confirmed by the reduction in a band size from colony PCR with the SOO632/SOO633 primers.

For the deletion of *XYL2* (*xyl2*∆)*,* the Re#22 strain expressing the pRS41N-Cas9 plasmid was co-transformed with the pRS42H-gRNA-XYL2.1 plasmid and the donor DNA fragments generated by the Kim064/Kim065 primers. The resulting transformants were selected on an YP agar plate containing 20 g/L galactose supplemented with both 100 μg/mL nourseothricin sulfate and 300 μg/mL hygromycin B. The deletion was confirmed by the reduction in a band size from colony PCR with the Kim066/Kim070 primers. Using the same method, the SR8 *xyl2*∆ strain was also constructed as a control strain. The resulting transformants were selected on YPD agar medium supplemented with both 100 μg/mL nourseothricin sulfate and 300 μg/mL hygromycin B.

**Promoter substitution of the *GLK1* gene by CRISPR/Cas9**

To create the SR8#22 *CYC1p_mGLK1,* SR8#22 *TEF1p_mGLK1,* and SR8#22 *CCW12p_mGLK1* strains, the donor DNA fragments were PCR-amplified from the genomic DNA of the D452-2 strain using the SOO668/SOO643 primers, the SOO669/SOO670 primers, or SOO639/SOO637 primers, respectively. The SR8#22 strain expressing the pRS41N-Cas9 plasmid was co-transformed with the pRS42K-gRNA-GLK1.2 plasmid and the prepared donor DNA fragments. The resulting transformants were selected on YP agar medium supplemented with 40 g/L xylose, 100 μg/mL nourseothricin sulfate, and 200 μg/mL G-418 sulfate. The promoter substitution was confirmed by Sanger sequencing of the colony PCR products with the SOO225/SOO641 primers.

**Construction of a hexokinase null mutant of SR8 (SR8∆3) by CRISPR/Cas9**

A quadruple auxotrophic mutant (*his*∆, *leu*∆, *trp*∆, *ura*∆) of the SR8 strain (SR8-4xAuxotroph) was created by CRISPR/Cas9 genome engineering as reported previously ([5](#_ENREF_5)). Next, the hexokinase null mutant (*glk1*∆, *hxk2*∆, *hxk1*∆) of the SR8 strain (SR8Δ3) was constructed as described above. First, the SR8-4xAuxotroph strain expressing the pRS41N-Cas9 plasmid was co-transformed with the pRS42H-gRNA-GLK1.1 plasmid and donor DNA fragments that are generated with the Jin3707/Jin3708 primers. The resulting transformants were selected on YPD agar medium supplemented with both 100 μg/mL nourseothricin sulfate and 150 μg/mL hygromycin B. The deletion (*glk1*∆) was confirmed by colony PCR using the Jin3709/Jin3710 primers. Next, *HXK2* was deleted using the pRS42K-gRNA-HXK2.2 plasmid and the Jin3767/Jin3768 primers, and the deletion was confirmed using the Jin3769/Jin3770 primers. Finally, *HXK1* was deleted using the pRS42H-gRNA-HXK1.2 plasmid and the Jin3762/Jin3763 primers, and the deletion was confirmed using the Jin3764/Jin3765 primers, yielding the SR8Δ3 strain.

**Doxycycline-controlled system of hexokinase expression in wild type D452-2**

The D452*∆*3i strain was also created using CRISPR/Cas9 genome engineering. The *HXK1* and *HXK2* genes were deleted as described above. For *GLK1* deletion*,* the donor DNA fragments were prepared using the Jin5143/Jin5144 primers to amplify the *MYO2p*-rtTA(S2)-*CYC1t* expression cassette from the pRS406-rtTA plasmid. In the resulting strain (D452*∆*3i), the *GLK1* gene was replaced with the MYO2p-rtTA(S2)-CYC1t expression cassette. This replacement was confirmed through colony PCR using the Jin5145/Jin5146 primers. Next, the pRS403-*tetO*_7_-*HXK2* plasmid or the pRS403-*tetO*_7_-*HXK1* plasmid was genome integrated into the *HIS3* locus of the D452*∆*3i strain, resulting in the D452∆3iHXK2 and the D452∆3iHXK1 strains, respectively.

**SI References**

1. Xu H, Kim S, Sorek H, Lee Y, Jeong D, Kim J, et al. PHO13 deletion-induced transcriptional activation prevents sedoheptulose accumulation during xylose metabolism in engineered Saccharomyces cerevisiae. Metabolic engineering. 2016;34:88-96.

2. DiCarlo JE, Norville JE, Mali P, Rios X, Aach J, Church GM. Genome engineering in Saccharomyces cerevisiae using CRISPR-Cas systems. Nucleic acids research. 2013;41(7):4336-43.

3. Goldstein AL, McCusker JH. Three new dominant drug resistance cassettes for gene disruption in Saccharomyces cerevisiae. Yeast. 1999;15(14):1541-53.

4. Hegemann JH, Heick SB, Pöhlmann J, Langen MM, Fleig U. Targeted gene deletion in Saccharomyces cerevisiae and Schizosaccharomyces pombe. Yeast Protocols. 2014:45-73.

5. Zhang G-C, Kong II, Kim H, Liu J-J, Cate JH, Jin Y-S. Construction of a quadruple auxotrophic mutant of an industrial polyploid Saccharomyces cerevisiae strain by using RNA-guided Cas9 nuclease. Applied and environmental microbiology. 2014;80(24):7694-701.

6. Taxis C, Knop M. System of centromeric, episomal, and integrative vectors based on drug resistance markers for Saccharomyces cerevisiae. Biotechniques. 2006;40(1):73.

7. Hegemann JH, Heick SB. Delete and repeat: a comprehensive toolkit for sequential gene knockout in the budding yeast Saccharomyces cerevisiae. Strain engineering: methods and protocols. 2011:189-206.

8. Hosaka K, Nikawa J-i, Kodaki T, Yamashita S. A dominant mutation that alters the regulation of INO1 expression in Saccharomyces cerevisiae. Journal of biochemistry. 1992;111(3):352-8.

9. Kim SR, Skerker JM, Kang W, Lesmana A, Wei N, Arkin AP, et al. Rational and evolutionary engineering approaches uncover a small set of genetic changes efficient for rapid xylose fermentation in Saccharomyces cerevisiae. PloS one. 2013;8(2):e57048.

**Supplementary Figures**


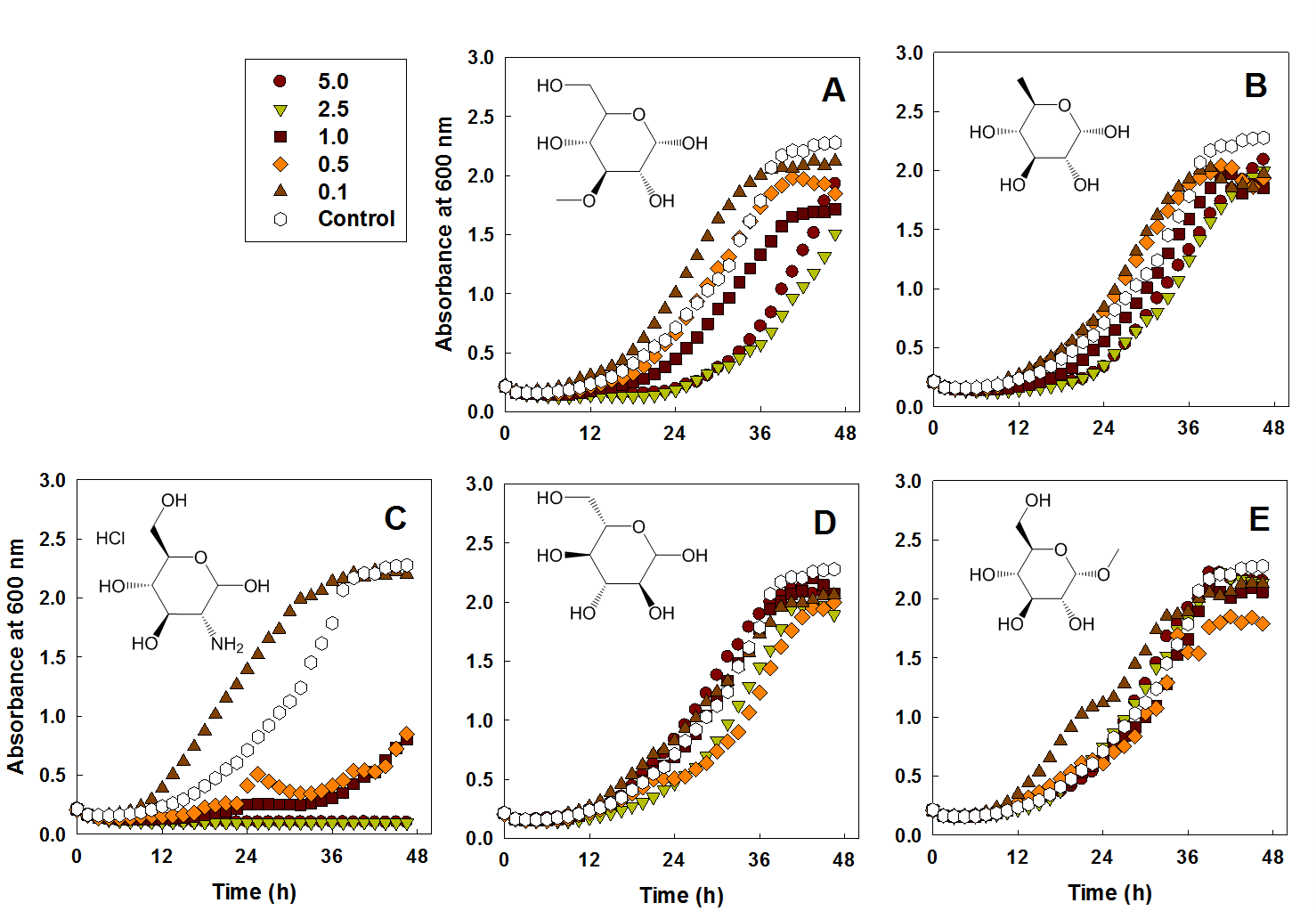


**Supplementary Figure S1.** Growth profiles of the SR8 strain in complex medium containing 40 g/L xylose and one of several glucose analogues at different concentrations: 3-O-methyl-D-glucopyranose (A), 6-deoxyglucose (B), glucosamine hydrochloride (C), L-glucose (E), and methyl α-D-glucopyranoside (E).

**Supplementary Figure S2.** Fermentation profiles of 40 evolved mutants isolated on a xylose plate (A) or a glucose plate (B). Based on the ability to grow using glucose as a sole carbon source (A and B), nine mutants were selected and evaluated for the ability to consume glucose and xylose simultaneously (C).

**Supplementary Figure S3.** Fermentation profiles of the parental strain SR8 (A and B) and the evolved strain SR8#22 (C and D) in complex medium with a single sugar: 40 g/L glucose (A and C) or 40 g/L xylose (B and D). (E) Comparison of sugar consumption rates in each condition.


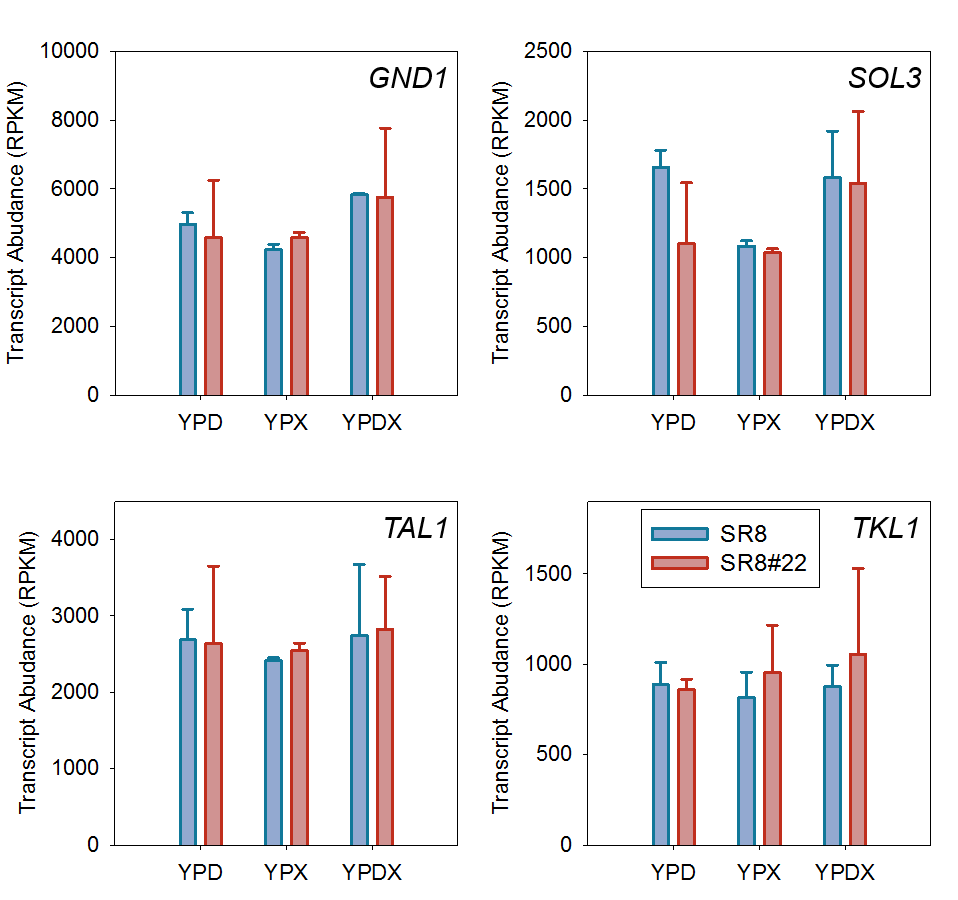


**Supplementary Figure S4.** Expression of pentose phosphate pathway genes with different carbon sources as determined by RNA-seq in the parental SR8 and evolved SR8#22. Expression profiles are shown of *GND1*, *SOL3*, *TAL1*, and *TKL1* genes. Cells were grown to mid-exponential phase and RNA was extracted and quantified using RNA-seq as described in materials and methods. RPKM: reads per kilobase of transcript per million mapped reads. YPD: glucose; YPX: xylose; YPDX: glucose and xylose mixture.

**Supplementary Figure S5.** Fermentation profiles of the SR8*mGLK1* (A) and the SR8*mGLK1mHXK2* (B) strains.


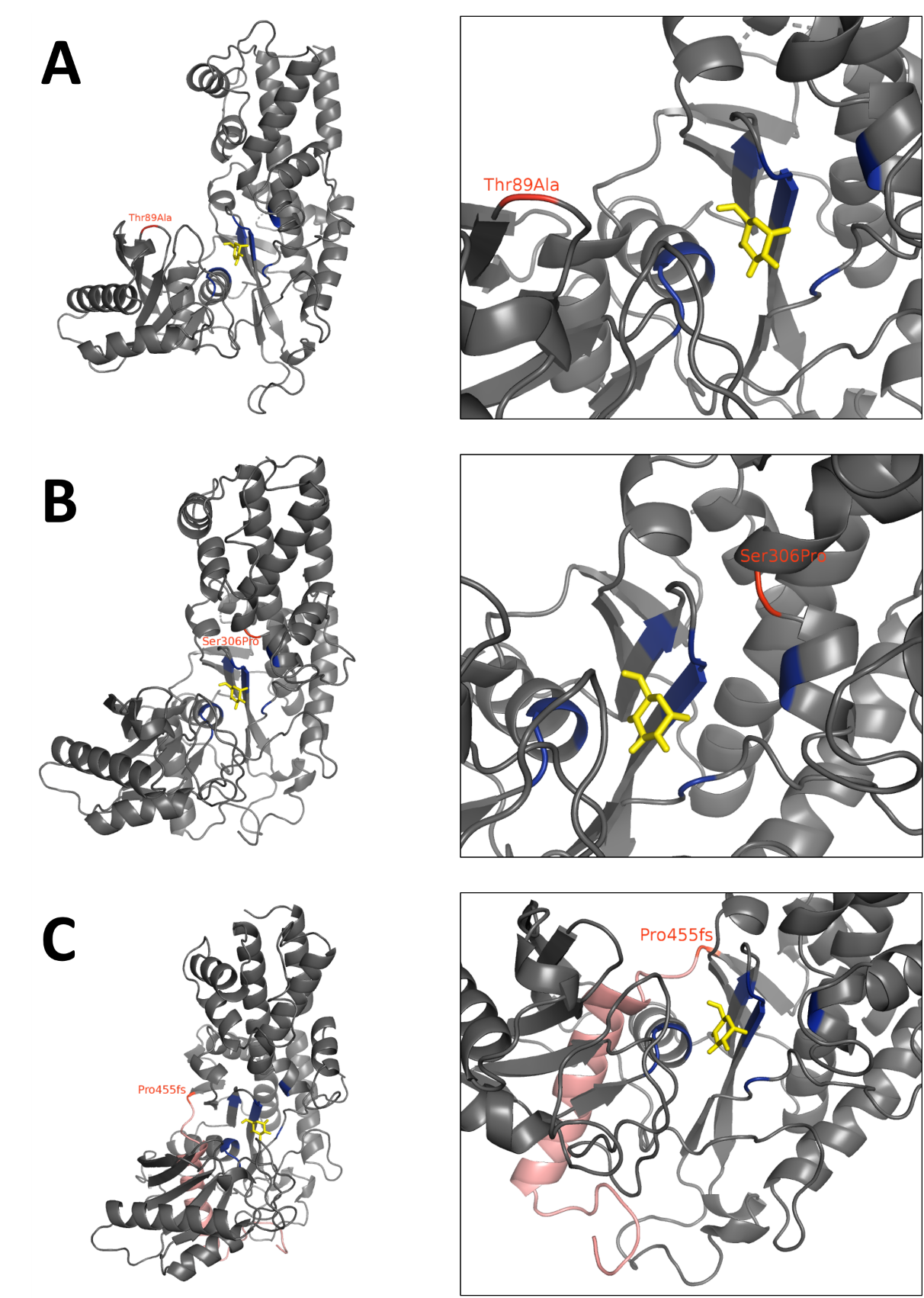


**Supplementary Figure S6.** Wild-type protein structures of Glk1p, Hxk1p, and Hxk2p with an enlarged view of the ligand-binding site relative to mutated residues. Site of mutations in the evolved SR8#22 strain are labeled in red ((A) Glk1p T89A; (B) Hxk1p S306P; (C) Hxk2p Pro455fs) while the predicted ligand-binding residues are labeled in blue. The residues C-terminal of the Hxk2p frameshift mutation in the evolved SR8#22 are labeled in pink.





**Supplementary Figure S7**. Comparison of hexokinase expression in the SR8 (blue) and SR8#22 (red) strains cultured with glucose, xylose, or a mixture of glucose and xylose. Cells were grown to mid-exponential phase and RNA was extracted and quantified using RNA-seq as described in materials and methods. A significant difference of P < 0.05 is indicated by a single asterisk while two asterisks indicates a stronger significance of P < 0.01. RPKM: reads per kilobase of transcript per million mapped reads. YPD: glucose; YPX: xylose; YPDX: glucose and xylose mixture.

**Supplementary Figure S8.** Fermentation profiles of the SR8#22 strain with a different hexokinase/glucokinase deletion (A-C) or the SR8#22 strain with a different *mGLK1* expression level (D-F): SR8#22 *glk1*Δ (A), SR8#22 *hxk2*Δ (B), SR8#22 *hxk1*Δ (C), SR8#22 *CYC1p*-*mGLK1* (low strength promoter, D), SR8#22 *TEF1p*-*mGLK1* (medium strength promoter, E), and SR8#22 *CCW12p*-*mGLK1* (high strength promoter, F) strains. Fermentations were performed in complex medium containing 40 g/L xylose and 40 g/L glucose under oxygen-limited conditions with initial OD 1.

**Supplementary Figure S9.** Fermentation profiles of SR8#22 with different single transporter deletions. Fermentation profiles of (A) SR8#22 *hxt2*∆, (B) SR8#22 *hxt3*∆, (C) SR8#22 *hxt4*∆, and (D) SR8#22 *hxt6/7*∆ in complex medium containing 40 g/L xylose and 40 g/L glucose under oxygen-limited conditions with initial OD 1.

**Supplementary Figure S10.** Co-consumption of glucose and xylose by limiting hexokinase activity. SR8Δ3 (hexokinase null mutant) with the inducible *HXK2* gene (A-G) was cultured in a mixture of glucose and xylose with various levels of doxycycline at (A) 0, (B) 2, (C) 4, (D) 6, (E) 8, (F) 10, and (G) 12 µg/mL. Fermentations were performed with 25 mL YP media in 125 mL flasks at an initial OD of 1.

**Supplementary Figure S11.** Modulating *HXK2* expression enables co-consumption of glucose and galactose. D452Δ3i (hexokinase null mutant) with the inducible *HXK2* gene was cultured in a mixture of glucose and galactose with various levels of doxycycline at (A) 0, (B) 2, (C) 4, (D) 6, (E) 8, (F) 10, and (G) 12 µg/mL. Fermentations were performed with 25 mL YP media in 125 mL flasks at an initial OD of 1.

**Supplementary Figure S12.** Modulating *HXK1* expression enables co-consumption of glucose and galactose. D452Δ3i (hexokinase null mutant) with the inducible *HXK1* gene was cultured in a mixture of glucose and galactose with various levels of doxycycline at (A) 0, (B) 2, (C) 4, (D) 6, (E) 8, (F) 10, and (G) 12 µg/mL. Fermentations were performed with 25 mL YP media in 125 mL flasks at an initial OD of 1.


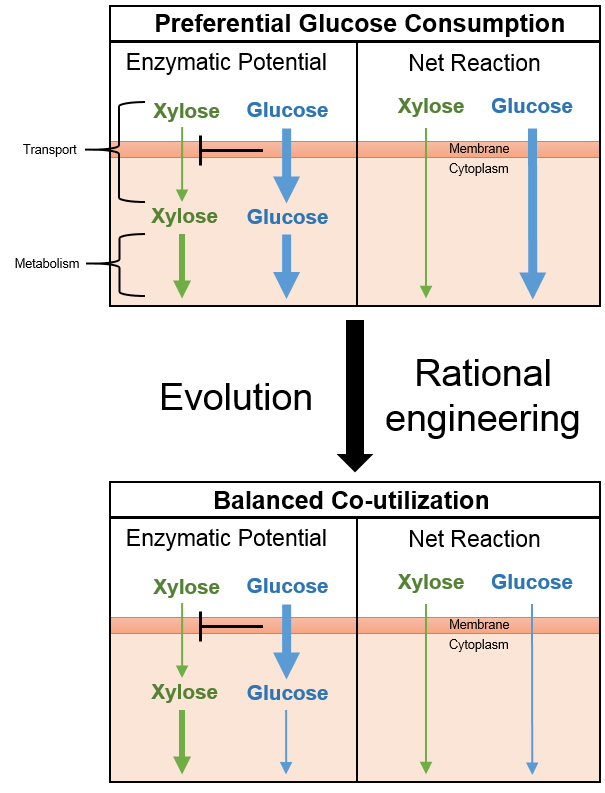


**Supplementary Figure S13.** Diagram outlining the model of balanced co-utilization presented in this study. Rapid transport of glucose and inhibition of xylose is counteracted by a reduced rate of intracellular glucose metabolism. By limiting the rate of glucose metabolism so that it matches the rate-limiting step of xylose metabolism in the presence of glucose (transport), balanced co-utilization and similar consumption rates for both sugars can be enabled.

**Supplementary Figure S14.** Enhanced xylitol production through co-assimilation of glucose and xylose. Fermentation profiles of (A) SR8 *xyl2*Δ and (B) Re#22 *xyl2*Δ cultured in a mixture of glucose and xylose.

**Supplementary Tables**

**Supplementary Table S1. Genotypes of the backcrossed recombinants**

|  | MAT | Phenotype | ***GLK1*** | ***HXK2*** | ***HXK1*** |
| --- | --- | --- | --- | --- | --- |
| SR8#22 | alpha |  | **265A>G** | **1364∆C** | **916T>C** |
| SR8#22-2 | A | SR8#22-like | **G** | **∆** | **C** |
| SR8#22-10 | A |  | **G** | **∆** | **C** |
| SR8#22-35 | A |  | **G** | **∆** | **C** |
| SR8#22-8 | alpha | SR8-like | A | **∆** | C |
| SR8#22-16 | alpha |  | A | C | T |
| SR8#22-19 | A |  | A | **∆** | C |

**Supplementary Table S2. Measurements of glucose/galactose co-fermentation characteristics of strains in this study**

| Strain | *r*_glucose_ (g/g/h) | *r*_xylose_ (g/g/h) | *P*_ethanol_ (g/g/h) | *Y*_ethanol_ (g/g) | Ethanol titer (g/L) |
| --- | --- | --- | --- | --- | --- |
| D452-2+403 | 2.33 ± 0.1 | N.D. | 0.95 ± 0.06 | 0.34 ± 0.01 | 17.37 ± 0.6 |
|  |  |  |  |  |  |
| D452∆3iHXK1 |  |  |  |  |  |
| [Dox] = 0 µg/mL | 0.14 ± 0.04 | 0.78 ± 0.03 | 0.32 ± 0.02 | 0.31 | 18.51 ± 0.29 |
| [Dox] = 2 µg/mL | 0.36 | 0.73 ± 0.01 | 0.41 ± 0.01 | 0.35 | 31.89 ± 1.11 |
| [Dox] = 4 µg/mL | 0.5 | 0.73 ± 0.01 | 0.5 | 0.36 | 32.70 ± 0.09 |
| [Dox] = 6 µg/mL | 0.61 ± 0.03 | 0.77 ± 0.04 | 0.58 ± 0.01 | 0.36 | 31.72 ± 0.53 |
| [Dox] = 8 µg/mL | 0.68 ± 0.06 | 0.71 ± 0.05 | 0.58 ± 0.02 | 0.36 | 33.41 ± 0.06 |
| [Dox] = 10 µg/mL | 0.73 ± 0.02 | 0.75 ± 0.01 | 0.59 | 0.37 | 33.64 ± 0.01 |
| [Dox] = 12 µg/mL | 0.80 ± 0.03 | 0.73 ± 0.02 | 0.64 | 0.36 | 33.41 ± 0.19 |
|  |  |  |  |  |  |
| D452∆3iHXK2 |  |  |  |  |  |
| [Dox] = 0 µg/mL | 0.03 ± 0.01 | 0.84 ± 0.02 | 0.34 | 0.29 ± 0.01 | 14.09 ± 0.3 |
| [Dox] = 2 µg/mL | 0.31 ± 0.03 | 0.72 ± 0.05 | 0.42 ± 0.01 | 0.34 | 28.61 ± 0.25 |
| [Dox] = 4 µg/mL | 0.51 | 0.72 ± 0.01 | 0.50 | 0.34 ± 0.01 | 31.68 ± 0.53 |
| [Dox] = 6 µg/mL | 0.55 ± 0.1 | 0.68 ± 0.12 | 0.55 ± 0.03 | 0.35 ± 0.01 | 32.28 ± 0.62 |
| [Dox] = 8 µg/mL | 0.70 ± 0.02 | 0.73 ± 0.01 | 0.61 | 0.37 | 33.65 ± 0.22 |
| [Dox] = 10 µg/mL | 0.75 ± 0.05 | 0.72 ± 0.05 | 0.63 ± 0.04 | 0.37 | 33.83 |
| [Dox] = 12 µg/mL | 0.92 ± 0.07 | 0.79 ± 0.09 | 0.65 ± 0.04 | 0.36 | 33.37 ± 0.32 |

Values are the average of biological duplicates with standard deviation. No standard deviation is shown when the value is below 0.01. *r*_glucose_, specific glucose consumption rate (g glucose/g dry cell weight/h); *r*_xylose_, specific xylose consumption rate (g xylose/g dry cell weight/h); *P*_ethanol_, specific productivity of ethanol (g ethanol/g dry cell weight/h); *Y*_ethanol_, ethanol yield (g ethanol/g consumed sugars). *r*_glucose_, *r*_xylose_, and *P*_ethanol_ are calculated in mid-exponential phase during co-consumption of glucose and xylose. *Y*_ethanol_ is calculated from the entirety of fermentation. Values were calculated from the fermentations shown in Figures 5, S11, and S12.

**Supplementary Table S3. Strains and Plasmids**

| **Plasmids** | **Description** | **Reference** |
| --- | --- | --- |
| pRS41N-Cas9 | A single-copy plasmid with a natMX marker expressing Cas9 | ([5](#_ENREF_5)) |
| pRS42K | A multi-copy plasmid with a kanMX marker | ([6](#_ENREF_6)) |
| pRS42H | A multi-copy plasmid with a hphMX marker | ([6](#_ENREF_6)) |
| pRS42K-gRNA | pRS42K with a specific guide RNA | This study |
| pRS42H-gRNA | pRS42H with a specific guide RNA | This study |
| pUG74 | An integrative plasmid with a natMX marker flanked with loxP | ([7](#_ENREF_7)) |
| pAG25 | An integrative plasmid with a natMX marker | ([3](#_ENREF_3)) |
| pRS406-rtTA | An integrative plasmid pRS406 with *MYO2p*-rtTA(S2)-*CYC1t* expression cassette | This study |
| pRS403-*tetO_7_* | An integrative plasmid pRS403 with a multicloning site between the *tetO_7_* promoter and *ADH1* terminator | This study |
| pRS403-*tetO_7_*-*HXK2* | An integrative plasmid pRS403 with *tetO_7_*-*HXK2*-*ADH1t* expression cassette | This study |
| pRS403-*tetO_7_*-*HXK1* | An integrative plasmid pRS403 with *tetO_7_*-*HXK1*-*ADH1t* expression cassette | This study |
| **Strains** |  |  |
| D452-2 | *MATα leu2 his3 ura3* | ([8](#_ENREF_8)) |
| SR8 | D452-2 expressing *Scheffersomyces stipitis* *XYL1*, *XYL2*, and *XYL3*, and evolved on xylose | ([9](#_ENREF_9)) |
| SR8#22 | SR8 evolved under the presence of 2-deoxyglucose and xylose | This study |
| SR8*mGLK1* | SR8 expressing *mGLK1* (265A>G) | This study |
| SR8*mGLKmHXK2* | SR8 expressing *mGLK1* and *mHXK2* (1364ΔC) | This study |
| Re#22 | SR8 expressing *mGLK1*, *mHXK2*, and *mHXK1* (916T>C) | This study |
| SR8#22 *mglk1*∆ | SR8#22 with *mGLK1* deletion | This study |
| SR8#22 *mhxk1*∆ | SR8#22 with *mHXK1* deletion | This study |
| SR8#22 *mhxk2*∆ | SR8#22 with *mHXK2* deletion | This study |
| SR8#22 *CYC1p-mGLK1* | SR8#22 with insertion of a *CYC1* promoter upstream of *mGLK1* gene | This study |
| SR8#22 *TEF1p-mGLK1* | SR8#22 with insertion of a *TEF1* promoter upstream of *mGLK1* gene | This study |
| SR8#22 *CCW12p-mGLK1* | SR8#22 with insertion of a *CCW12* promoter upstream of *mGLK1* gene | This study |
| SR8#22 *hxt2*∆ | SR8#22 with *HXT2* deletion | This study |
| SR8#22 *hxt3*∆ | SR8#22 with *HXT3* deletion | This study |
| SR8#22 *hxt4*∆ | SR8#22 with *HXT4* deletion | This study |
| SR8#22 *hxt6/7*∆ | SR8#22 with *HXT6/7* deletion | This study |
| SR8-4xAuxotroph | SR8 *leu2 his3 ura3 trp1* | This study |
| SR8*∆*3 | SR8-4xAuxotroph with deletions in *HXK1 HXK2* and *GLK1* | This study |
| SR8*∆*3iHXK2 | SR8*∆*3 with pRS406-rtTA and pRS403-*tetO_7_*-*HXK2* | This study |
| D452-2+403 | D452-2 with pRS403 | This study |
| D452*∆*3i | D452-2 with deletions in *HXK1, HXK2,* and *glk1∆*::*MYO2p*-*rtTA(S2)*-*CYC1t* | This study |
| D452*∆*3iHXK1 | D452-2*∆*3i with pRS403-*tetO_7_*-*HXK1* | This study |
| D452*∆*3iHXK2 | D452-2*∆*3i with pRS403-*tetO_7_*-*HXK2* | This study |
| SR8 *xyl2*∆ | Xylitol-producing SR8 with a deletion in the *XYL2* gene | This study |
| Re#22 *xyl2*∆ | Xylitol-producing Re#22 through a deletion in the *XYL2* gene | This study |

**Supplementary Table S4. Guide RNA structure**

| Description | Sequence |
| --- | --- |
| *SNR52* promoter | tctttgaaaagataatgtatgattatgctttcactcatatttatacagaaacttgatgttttctttcgagtatatacaaggtgattacatgtacgtttgaagtacaactctagattttgtagtgccctcttgggctagcggtaaaggtgcgcattttttcacaccctacaatgttctgttcaaaagattttggtcaaacgctgtagaagtgaaagttggtgcgcatgtttcggcgttcgaaacttctccgcagtgaaagataaatgatc |
| Target sequences |  |
| GLK1.1 | AACAGAACATATACGGAAAT |
| GLK1.2 | TGATAGAGTTGTATTAGTGG |
| HXK2.1 | GTTCCTGCTGAAGATGGTTC |
| HXK2.2 | TCAAAGCAACAACTTCAA |
| HXK1.1 | TTCACCCAAGTAGTAACCGG |
| HXK1.2 | TAGTTTATACTTGGATTGAG |
| HXT2.1 | CAAAGCCAATCGCCGCATAT |
| XYL2.1 | aatgactgctaacccttcct |
| Structural crRNA | GTTTTAGAGCTAGAAATAGCAAGTTAAAATAAGGCTAGTCCGTTATCAACTTGAAAAAGTGGCACCGAGTCGGTGGTGC |
| *SUP4* terminator | tttttttgttttttatgtct |

**Supplementary Table S5. Primers**

| Names | Description | Sequences |
| --- | --- | --- |
| For donor DNA preparation for Cas9 engineering | | |
| SOO459 | *mGLK1*_F | GGAGCGCGGTGTTTTACTAGCCGCCGACCTGGGTGGTGCTAATTTCCGTATATGTTCTGT |
| SOO460 | *mGLK1*_R | CATTTGCTCCATGGAGAAAGTATGATCTCCATGCAAGTTAACAGAACATATACGGAAATT |
| SOO510 | *mHXK2*_F | GACGACTACCCAATCAAGATTGTTCTGCTGAAGATGGTTCCAGTGCTGGTGCCGCTGTTA |
| SOO511 | *mHXK2*_R | GACTTACCTTCAGCAATTCTTTTTTGGGCCAAAGCAGCAATAACAGCGGCACCAGCACtG |
| SOO546 | *mHXK1*_F | ATCTCCAAGACCTGGTCAACAAGCTTTTGAAAAGATGACTCCAGGTTACTACTTGGGTGA |
| SOO547 | *mHXK1*_R | GCCCTTCTCGTTTAATTCAAGTAACACTAGACGCAACAATTCACCCAAGTAGTAACCTGG |
| Jin3266 | *hxt2∆*_F | TCTCAATTCCTCTTATATTAGATTATAAGAACAACAAATTAAATTACAAAAAGACTTATAAAGCAACATACGAGCGACTCGATGATCAAC |
| Jin3267 | *hxt2∆*_R | GAAGATCATCTATTAAAGTATTAGTAGCCATTAGCCTTAAAAAAATCAGTGCTAGTTTAAGTATAATCTCGTTGATCATCGAGTCGCTCG |
| Kim064 | *xyl2*∆_F | aattatctactttttacaacaaatataaaa GGCTATAGCTTGAAGCTGTA |
| Kim065 | *xyl2*∆_R | tttcaattcaattcaatttactcagggccg TACAGCTTCAAGCTATAGCC |
| SOO668 | *CYC1p*_*mGLK1_*F | CCCCCCCATCAGTGCCCAACTCAGCTTCCGTAAACCACAACAAAAGCGCCAGTTCATTTG |
| SOO643 | *CYC1p*_*mGLK1*_R | TCTCTCAGTGGCTTTGTGTAAGTCGTCGAATGACATGTGTGTATTTGTGTTTGTGTG |
| SOO669 | *TEF1p_mGLK1*_F | CCCATCAGTGCCCAACTCAGCTTCCGTAAACCACAACAAATGTTTCTACTCCTTTTTTAC |
| SOO670 | *TEF1p_mGLK1*_R | CTCAGTGGCTTTGTGTAAGTCGTCGAATGACATTTTGTAATTAAAACTTAGATTAGATTG |
| SOO639 | *CCW12p_mGLK1*_F | GCGTAACAAAATATATATATATATATATATATATATGTATGTCACGCAAAAGAAAACCTT |
| SOO637 | *CCW12p_mGLK1*_R | TCTCTCAGTGGCTTTGTGTAAGTCGTCGAATGACATTATTGATATAGTGTTTAAGCGAAT |
| Jin3707 | *glk1∆*_F | ccccatcagtgcccaactcagcttccgtaaaccacaacaccaccactaatacaactctatcatacacaag TAAGGCGAGCTCATACCGTC |
| Jin3708 | *glk1∆*_R | tatatatataaaggagagaagatggtaagtacggtgggatacgtacacaaaccaaaaaaatgtaaaaaga GACGGTATGAGCTCGCCTTA |
| Jin3767 | *hxk2∆*_F | ttcgctttttctttgaaaaggttgtaggaatataattctccacacataataagtacgctaattaaataaa ACGACCGACGTACGATTCAA |
| Jin3768 | *hxk2∆*_R | tagaaaacatgttcacataagtagaaaaagggcaccttcttgttgttcaaacttaatttacaaattaagt TTGAATCGTACGTCGGTCGT |
| Jin3762 | *hxk1∆*_F | actcaattagaattcttttcttttaatcaaactcacccaaacaactcaattagaatactgaaaaaataag GTGTAACTCAGATGAGCTAC |
| Jin3763 | *hxk1∆*_R | ggcatcactcataagaataataatattaagggagggaaaaacacatttatatttcattacatttttttca GTAGCTCATCTGAGTTACAC |
| Jin5143 | *glk1::rtTA*_F | AAATTTTAGACGCGGCGCTTGCACCCCGCATTATAAGTGGTG ctcaagcaaggttttcag |
| Jin5144 | *glk1::rtTA*_R | TACCGGTACCGAAAATACATCCGATGACCGGCTCCGAG gaattccacttaatgtatcaac |
| For sequencing confirmation of Cas9 engineering | | |
| SOO8 | *GLK1*_F | GGCGGATCCATGTCATTCGACGACTTACACAA |
| SOO9 | *GLK1*_R | GGCGTCGACTCATGCTACAAGCGCACACA |
| SOO170 | *HXK2*_F | GGCGGATCCTTAGCACTACTGGGACAAGC |
| SOO171 | *HXK2*_R | GGCGCGGCCGCGAGGAAGTGTAGAGAGGGTT |
| SOO4 | *HXK1*_F | GCCGGATCCCACCTGGTCTTACCTCGAAC |
| SOO5 | *HXK1*_R | GCCGCGGCCGCCGACTTTCTCCCTCTCTCCA |
| SOO225 | *GLK1* upstream_F | TATAAGTGGTGTGCCGACG |
| SOO641 | *GLK1* internal_R | GATGACCGCTCTCTCAGTGG |
| Jin3709 | *GLK1* uptream_F | taccaattagacatgctgcttgc |
| Jin3710 | *glk1∆*_R | GACGGTATGAGCTCGCCTTA |
| Jin3769 | *HXK2* upstream_F | GCTCCAGAGCTCCACATTG |

**Supplementary Table S5. continued**

| Names | Description | Sequences |
| --- | --- | --- |
| For sequencing confirmation of Cas9 engineering | | |
| Jin3770 | *hxk2∆*_R | TTGAATCGTACGTCGGTCGT |
| Jin3764 | *HXK1* upstream_F | GCCAGATCTCAGTATAGCAG |
| Jin3765 | *hxk1∆*_R | GTAGCTCATCTGAGTTACAC |
| Jin5145 | *glk1::rtTA*_Confirm_F | GTGTCACTAGGTGCAATTGCC |
| Jin5146 | *glk1::rtTA*_Confirm_R | CGACAGCACTTCGGATGA |
| For gene deletion using a drug resistance marker | | |
| SOO252 | *glk1::loxP*_F | CCCCATCAGTGCCCAACTCAGCTTCCGTAAACCACAACACCACCACTAATACAACTCTATCATACACAAG CAGCTGAAGCTTCGTACGC |
| SOO253 | *glk1::loxP*_R | TATATATAAAGGAGAGAAGATGGTAAGTACGGTGGGATACGTACACAAACCAAAAAAATGTAAAAAGA GCATAGGCCACTAGTGGATCTG |
| Jin2335 | *hxt3::loxP*_F | AATAGAATCACAAACAAAATTTACATCTGAGTTAAACAATC CAGCTGAAGCTTCGTACGC |
| Jin2336 | *hxt3::loxP*_R | AAATACACTATTATTCAGCACTACGGTTTAGCGTGAAA GCATAGGCCACTAGTGGATCTG |
| Jin2337 | *hxt4::loxP*_F | GGTTTGGTTTTGAAACACTTTTACAATAAAATCTGCCAAAA CAGCTGAAGCTTCGTACGC |
| Jin2338 | *hxt4::loxP*_R | ATTCCTTGAAGGAAGTCTATATTATTTAATTAACTGAC GCATAGGCCACTAGTGGATCTG |
| SOO401 | *hxt6/7::loxP*_F | AACATATAAAAAGAGCTCGAGAAAAGACATATGGTTTGTAACTATCTTCTTCTTTTTTCCAATTTTTCTGT CAGCTGAAGCTTCGTACGC |
| SOO406 | *hxt6/7::loxP*_R | TTCTGAGAACAAATGATCAAAAACTTGAAAATTAAACTGTATTATTTTGTATATATTAAAAACGTATT GCATAGGCCACTAGTGGATCTG |
| SOO179 | *hxk2∆*_F | GCTTTTTCTTTGAAAAGGTTGTAGGAATATAATTCTCCACACATAATAAGTACGCTAATTAAATAAAATG CGTACGCTGCAGGTCGAC |
| SOO180 | *hxk2∆*_R | AAAACATGTTCACATAAGTAGAAAAAGGGCACCTTCTTGTTGTTCAAACTTAATTTACAAATTAAGTTTA ATCGATGAATTCGAGCTCG |
| SOO142 | *hxk1∆*_F | CAATTAGAATTCTTTTCTTTTAATCAAACTCACCCAAACAACTCAATTAGAATACTGAAAAAATAAGATG CGTACGCTGCAGGTCGAC |
| SOO143 | *hxk1∆*_R | ATCACTCATAAGAATAATAATATTAAGGGAGGGAAAAACACATTTATATTTCATTACATTTTTTTCATTA ATCGATGAATTCGAGCTCG |
| For deletion confirmation | | |
| SOO148 | natMX_R | GGATGTATGGGCTAAATG |
| SOO187 | natMX_R | GTGAAGGACCCATCCAGT |
| SOO225 | *GLK1* uptream_F | TATAAGTGGTGTGCCGACG |
| Jin2343 | *HXT3* uptream_F | ATTCGGTTAAACTCTCGG |
| Jin2344 | *HXT4* upstream_F | TTCATGAAAAATTCCAGAGT |
| SOO407 | *HXT6* upstream_F | GCGCCAAGACAAATGTTTC |
| SOO21 | *HXK2* uptream_F | AAATGGCTATCATGCCGGAC |
| Jin687 | *HXK1* uptream_F | CCCGTTTGTTGGAAGATAGC |
| SOO632 | *HXT2* upstream_F | GTTGACAGGTCAGTTAAGGCACAG |
| SOO633 | *HXT2* downstream_R | GTTGATCATCGAGTCGCTCG |
| Kim066 | *XYL2* upstream_F | ACCGTCGCTCGTGATTTG |
| Kim070 | *XYL2* downstream_R | GAGTGTGAAGCCAAGAAGG |
